# Molecular evidence confirms occurrence of *Rhipicephalus microplus* Clade A in Kenya and sub-Saharan Africa

**DOI:** 10.1186/s13071-020-04266-0

**Published:** 2020-08-27

**Authors:** Esther G. Kanduma, David Emery, Naftaly W. Githaka, Edward K. Nguu, Richard P. Bishop, Jan Šlapeta

**Affiliations:** 1 Present Address: Department of Biochemistry, School of Medicine, University of Nairobi, P.O. Box 30197-00100, Nairobi, Kenya; 2 Sydney School of Veterinary Science (SSVS), University of Sydney, Sydney, NSWs Australia; 3 International Livestock Research Institute (ILRI), P.O. Box 30709-00100, Nairobi, Kenya; 4 Department of Veterinary Microbiology and Pathology, Washington State University, Pullman, USA

**Keywords:** Ticks, *Rhipicephalus decoloratus*, *Rhipicephalus*, Babesiosis, *Babesia bovis*, *cox*1, Mitochondrial genome, Genetic differentiation, Phylogenetics

## Abstract

**Background:**

The tick vector *Rhipicephalus microplus* which transmits *Babesia* spp. and rickettsial pathogens has not been reported in Kenya since 1998. More recently, the pathogenic *Babesia bovis* has been detected in cattle blood DNA. The status of *R. microplus* in Kenya remains unknown. This study employed morphological and molecular tools to characterize *R. microplus* originating from Kenya and assess the genetic relationships between Kenyan and other African *R. microplus* genotypes.

**Methods:**

Ticks were collected in south-eastern Kenya (Kwale County) from cattle and characterized to investigate the existence of *R. microplus*. Genetic and phylogenetic relationships between the Kenyan and other annotated *R. microplus* reference sequences was investigated by analysis of the cytochrome *c* oxidase subunit 1 (*cox*1) gene. To further characterize Kenyan ticks, we generated low coverage whole genome sequences of two *R. microplus*, one *R. decoloratus* and *R. appendiculatus*. A *B. bovis* specific TaqMan probe qPCR assay was used to detect *B. bovis* in gDNA from *R. microplus* ticks.

**Results:**

Occurrence of *R. microplus* was confirmed in Kwale County, Kenya. The Kenyan *R. microplus cox*1 sequences showed very high pairwise identities (> 99%) and clustered very closely with reference African *R. microplus* sequences. We found a low genetic variation and lack of geographical sub-structuring among the African *cox*1 sequences of *R. microplus*. Four complete mitochondrial (mt) genomes for two *R. microplus*, one *R. decoloratus* and one *R. appendiculatus* were assembled from next generation sequence data. The mitochondrial genome sequences of the two Kenyan *R. microplus* ticks clustered closely with reference genome sequences from Brazil, USA, Cambodia and India forming *R. microplus* Clade A. No *B. bovis* was detected in the Kwale *R. microplus* DNA.

**Conclusions:**

These findings confirm the presence of *R. microplus* in Kenya and suggest that *R. microplus* Clade A is prevalent in cattle in sub-Saharan Africa. These and other recent findings of widespread occurrence of *R. microplus* in Africa provide a strong justification for urgent surveillance to determine and monitor the spread of *R. microplus* and vector competence of *Boophilus* ticks for *B. bovis* in Africa, with the ultimate goal of strategic control.
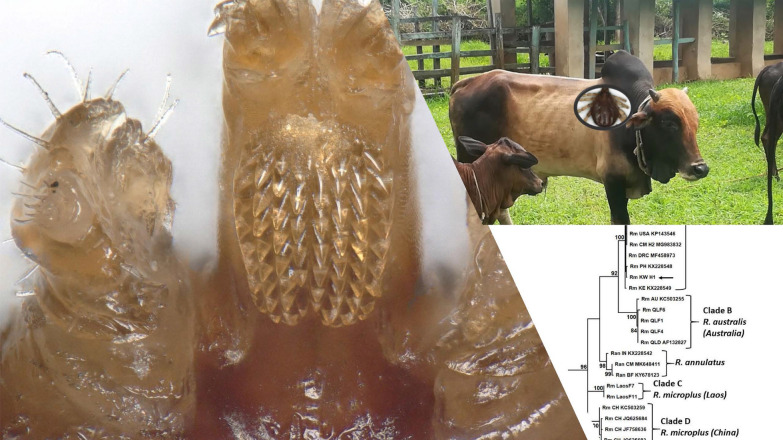

## Background

The Asian blue tick, *Rhipicephalus microplus*, has a widespread distribution in many tropical and subtropical areas of Asia, north-eastern Australia, South and Central America, southern and eastern Africa [1]. Historically, *R. microplus* has been known to occur in East and South Africa since the second wave of cattle introduction into Africa following the rinderpest epidemic in 1896 when *R. microplus*-infested cattle were imported from southern Asia *via* Madagascar [2]. In the last decade it has been reported in many West African countries [3–8] and more recently in Angola [9], Cameroon [10] and Uganda [11].

*Rhipicephalus microplus* is considered more economically important than other African ticks including *R. decoloratus* and *R. annulatus* because of its invasive capabilities and higher competence for *Babesia bovis*, the causative agent for a fatal form of bovine babesiosis [12]. In addition to pathogen transmission, the tick also causes direct economic losses to productivity and through hide damage [13]. The spread of *R. microplus* to new areas in Africa is a big threat to livestock industries and livelihoods of rural populations, who often depend on livestock for their survival. Annual economic losses associated with babesiosis and anaplasmosis in Kenya was estimated at about $6.9 million per year [14] and 70% of cattle are assumed to be at risk. The economic importance of *R. microplus* is also compounded by its propensity to displace other tick species, its higher vectoral capacity and ability to accumulate resistance to acaricides [15]. Already, widespread acaricide resistance has been reported in West Africa, where the tick is spreading [3, 5].

*Rhipicephalus microplus* was first recorded in Kenya in 1974 [16] and then later in 1998 [17] within a very limited area around Kwale County along the Kenyan Coast. However, more recently, *B. bovis* has been detected in cattle blood in central and western Kenya [18, 19], strongly suggesting the presence of its vector, *R. microplus.* In order to prevent the spread of *B. bovis*, validated methods to identify *R. microplus* are required, discriminating between this species and the endemic *R. decoloratus* and other *Boophilus* ticks. Based on cytochrome *c* oxidase subunit 1 (*cox*1) and mitochondrial genome phylogenetics, five distinct geographical clusters of the *R. microplus* species complex have been reported [20, 21]. They include Clade A (Africa, Asia and South America) and Clade B (southern China and northern India) of Burger et al. [20]; Clade C (Malaysia and India) of Low et al. [21], *R. australis* and *R. annulatus*. The Clade B lineage which was found to be more closely related to *R. annulatus* than to ticks in Clade A or C is thought to constitute a cryptic species restricted to China and parts of India [20].

Therefore, to determine if *R. microplus* does exist in Kenya, this study employed morphological and molecular tools to characterize and genotype tick specimens collected from Kwale County. Ticks were genetically and morphologically characterized to deliver reference material for the region. The reference material was characterised using scanning electron microscopy, digital microscopy, sequencing of *cox*1 as well as analysis of complete mtDNA from whole genome sequence data. DNA isolated from ticks was also tested for the presence of the pathogenic *B. bovis* DNA.

## Methods

### Study site

**Fig. 1:**
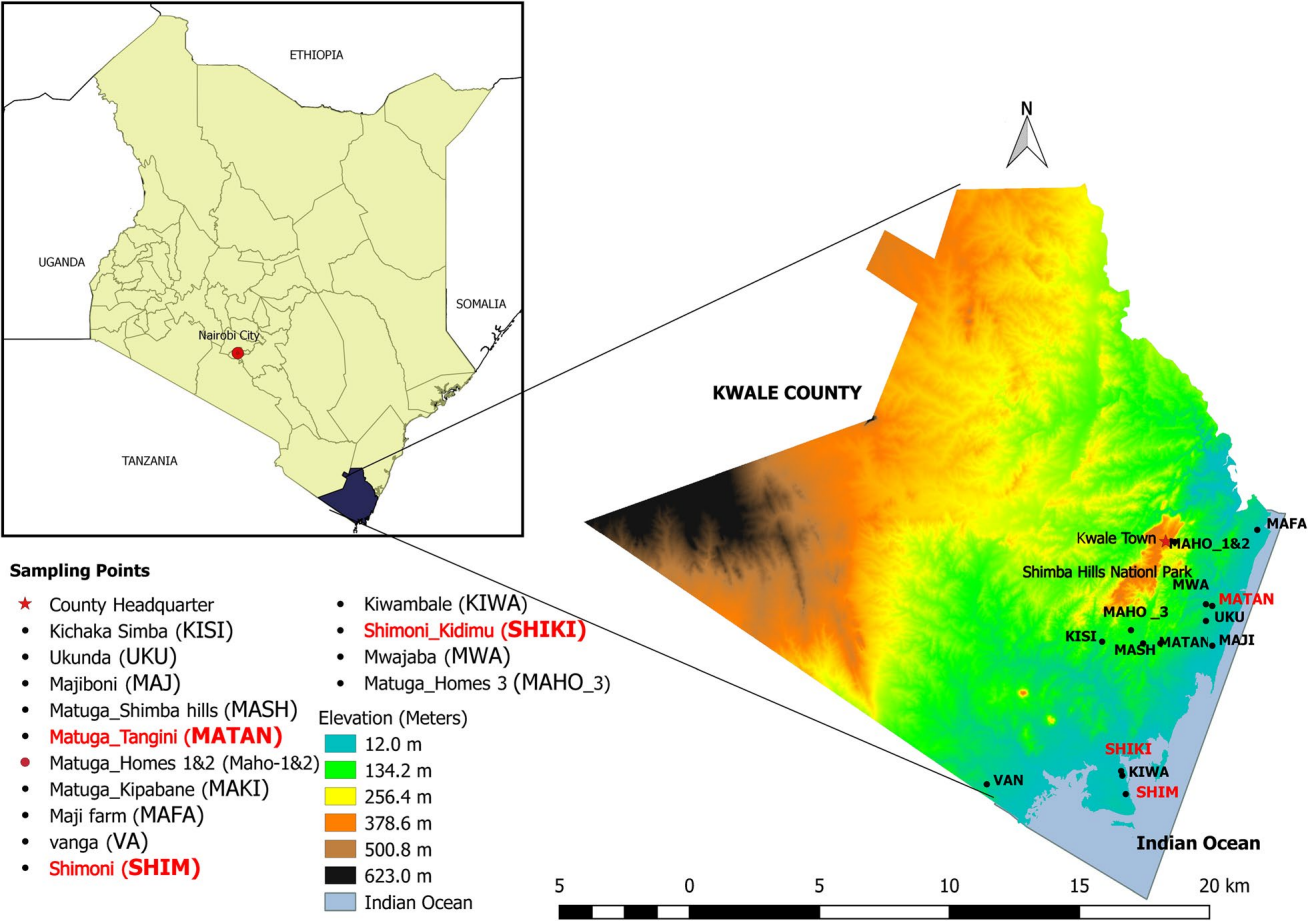
Map of Kenya showing Kwale County. Localities shown on the map were sampled for ticks. The tick samples analysed in this study were mainly collected from Matuga Tangini, Shimoni, Shimoni Kidimu and several other unspecified sites in the County

Tick samples were collected in May 2019 from cattle herds in several sites in Kwale County, Kenya (Fig. 1, Additional file 1: Table S1). Kwale County has an area of 8270.3 km^2^ and borders Indian Ocean to the East and South-East and Tanzania to the South-West. It lies between latitudes 30.05° to 40.75° South and longitudes 38.52° to 39.51° East. The County has a coastal plain that lies 30 m above sea level after which there is a foot plateau at an altitude of between 60–135 m above sea level. A coastal range characterized by hills rises steeply from the foot plateau to an altitude between 150–462 m above sea level. The final zone is a semi-arid plateau that stands at an altitude of about 180–300 m above sea level on the western boundary of the County. The County has a tropical type of climate influenced by monsoon seasons. The average temperature is about 23 °C with a maximum temperature of 25 °C being experienced in March and minimum temperature of 21 °C experienced in July. On average, annual precipitation in the County is less than 800 mm. Rainfall is bi-modal with a short rainy season from October to December and a long rainy season from April to July. There is a strong east to west gradient of decreasing precipitation with eastern (coastal) parts of the County receiving greater than 1000 mm of precipitation per year, while a majority of the central to west areas receive around 500–750 mm. Some areas along the western side of the County receive less than 500 mm of precipitation per year.

### Tick samples

Live adult ticks were plucked directly from cattle by use of steel forceps and placed in tubes. A total of 35 ticks, all from localities in Kwale County [Kwale Kidimu (*n* = 6), Matuga Tangini (*n* = 6), Shimoni Kidimu (*n* = 5) and unspecified locations in Kwale County (*n* = 18)] (Additional file 1: Table S1) were subjected to morphological and molecular analyses. In Kenya, *R. decoloratus* is endemic and widely distributed and is likely to be confused with *R. microplus*. Thus, reference *R. microplus* and *R. decoloratus* ticks were compared to the Kwale ticks. The 11 reference specimens included three *R. australis* from the Tick Fever Centre, Queensland, Australia, two *R. microplus* and two *R. decoloratus* from Cameroon, two *R. microplus* from Laos and two *R. decoloratus* from a laboratory colony maintained at the International Livestock Research Institute (ILRI), Nairobi (Additional file 1: Table S1). Since most of the ticks were semi- or fully engorged and some had missing mouth parts, only those specimens that were intact could be tentatively identified based on morphological features described in the identifications guides and keys used.

### Morphological identifications

Key morphological features of the tick specimens were observed under a compound microscope following standard keys and identification guides in Walker et al. [22] and Barker & Walker [23]. Images of key characteristic features were captured using a digital microscope (VHX-6000, KEYENCE Inc., Japan). Scanning electron microscopy (SEM) analysis of some reference specimens was done. Whole tick specimen was adhered to a mounting SEM stub (Ted Pella Inc., USA) with a double-sided carbon tape. The stub with the tick was then placed on a rotary planetary specimen stage within a K550X Sputter Coater unit (Quorum Technologies, Kent, UK) and coated with gold (Au) using the following parameters: current 25 mA, time 2:00 min and coating 15 nm. Once coated, the specimen was placed in a JEOL Neoscope, JCM-6000 (JEOL Inc., Nikon Inc., Japan) for imaging.

### DNA isolation, PCR amplification and sequencing of the *cox*1 gene

Using sterile single-use scalpel blades, 3–4 cuts were made on the body of the tick sparing key features important in morphological identifications. All semi- and fully engorged ticks were cut into two halves along the abdomen. DNA was extracted using the ISOLATE II Genomic DNA Kit (Bioline, Sydney, Australia) following the manufacturer’s protocol. Total DNA was eluted into 70 µl of elution buffer (Tris buffer, pH = 8.5, preheated to 70 °C). A 604 nucleotide 5′-fragment of the cytochrome *c* oxidase subunit 1 (*cox*1) gene was amplified in a conventional PCR using the forward primer S0725 (F1) (5′-TAC TCT ACT AAT CAT AAA GAC ATT GG-3′) and reverse primer S0726 (R1) (5′-CCT CCT CCT GAA GGG TCA AAA AAT GA-3′) [24]. MyTaq™ Red Mix (Bioline, Sydney, Australia) was used for *cox*1 amplification in 25 μl reactions using 1 μl of each of the two primers (10 pmol) and 2 μl template DNA. The PCR run conditions were: initial denaturation at 95 °C for 5 min, followed by 34 cycles of 94 °C for 10 s, 55 °C for 10 s and 72 °C for 15 s, and a final extension step at 72 °C for 7 min. A positive control and a negative no-template water control were included in all the reactions which were performed in a T100™ Thermal Cycler (Bio-Rad, Australia). PCR products were purified and sequenced at Macrogen Ltd (Seoul, South Korea).

### Sequencing and assembly of mitochondrial DNA from whole genome sequencing data

Isolated genomic DNA was used for NEBNext® DNA Library preparation following manufacturer’s recommendations. Indices were added to each of the four samples sequenced followed by the next-generation sequencing using 150-bp paired-end Illumina HiSeq 2500 sequencing systems utilizing a depth of 1 Gb of raw sequence data (Novogene, Singapore). The complete mitochondrial genome (mtDNA) of the four specimens comprising of two *R. microplus*, one *R. decoloratus* and one *R. appendiculatus* was assembled from FastQ data using the MITObim pipeline available at https://github.com/chrishah/MITObim with the complete mtDNA sequence of *R. microplus* (KC503260) as bait. The assembly was repeated three times with varying percentage of the raw FastQ sequence data used (10–50%), keeping mtDNA coverage at 60–100×. The obtained mtDNA was annotated with the aid of MITOS Web Server available at http://mitos.bioinf.uni-leipzig.de/ and aligned with available *Rhipicephalus* species genomes.

### TaqMan qPCR assay for detection of mammalian and *Babesia bovis* DNA

A TaqMan qPCR assay targeting a mammalian housekeeping gene, glyceraldehyde-3-phosphate dehydrogenase (GAPDH) [25] was conducted to confirm the presence of mammalian DNA in the Kwale *R. microplus* DNA samples. To detect *B. bovis* DNA, an assay targeting two different *B. bovis-*specific genes was performed. One set targeted the nuclear *18S* rDNA [26] and the other targeted the mitochondrial cytochrome *b* gene [27]. The primer and probe sequences used for detection of both mammalian and *B. bovis* DNA are listed in Table 1. The qPCR was conducted in a CFX96 Touch™ Real-Time PCR detection system (Bio-Rad, Australia). The 20 µl reactions included 10 µl of SensiFAST 2× Probe Mix (Bioline, Sydney, Australia), 0.8 µl of each oligonucleotide primer, 0.2 µl of the FAM or HEX labelled probe, and 2 µl of genomic DNA template. Temperature cycling conditions were: 95 °C for 3 min followed by 39 cycles of 95 °C for 10 s, 54 °C for 15 s and 72 °C for 30 s. The sensitivity and efficiency of the *B. bovis* assay was determined by using serial 10-fold dilutions of *B. bovis* control DNA (48 ng/µl) ranging from 1:10–1:10^7^. Positive and negative controls (no-template PCR grade water) were included in each PCR run. The threshold was set to 100 relative fluorescence units (RFUs) for the three assays and the cycle quantification (Cq) scores corresponding to the PCR cycle number at which the amplification curve of each sample intersected the threshold line were recorded for each sample.

**Table 1:** Details of primers used for mammalian DNA and *B. bovis* qPCR detection assays

Target gene	Primer/probe name	Sequence	Size (bp)	References
*18S*	Reverse: S0933_BoR_18S	AGTCGTGCGTCATCGACAAA	20	Kim et al. [26]
*18S*	Forward: S0934_BoF_18S	AGCAGGTTTCGCCTGTATAATG	22	
*18S*	Probe (5′-FAM-3′): S0935_BoP	CCTTGTATGACCCTGTCGTACCGTTGG	27	
Cytochrome *b*	Forward: S0936_bovisF160_Cytb	ATATGTTTGCATTTGCTG	18	Zhang et al. [27]
Cytochrome *b*	Reverse: S0937_bovisR249_cytb	CTCCAAACCAATATGAAAG	19	
Cytochrome *b*	Probe (5′-HEX-3′): S0938_bovis_cytb	CAAACCATAAAGTCATCGGTATATCCTAC	29	
*GAPDH*	Forward: S0631_Dog_F	TCAACGGATTTGGCCGTATTGG	22	Nijhof et al. [25]
*GAPDH*	Reverse: S0634_Dog_R	TGAAGGGGTCATTGATGGCG	20	
*GAPDH*	Probe (5′-HEX-3′): S0632_Dog	CAGGGCTGCTTTTAACTCTGGCAAAGTGGA	30	

*Note*: The fluorescence label of the Taqman probes is shown in brackets

### Data analysis

*Cox*1 sequence chromatograms were visually inspected and resulting sequences edited manually using CLC Main Workbench 20 software (CLC bio, Qiagen GmbH, Hilden, Germany). Sequences were trimmed to remove low quality reads at the 5′- and 3′-ends and consensus sequences generated from the sequenced fragments. Molecular identity of the study ticks was confirmed *via* BLASTN [28] searches of the *cox*1 against the GenBank’s non-redundant nucleotide sequence database. Multiple sequence alignments of the *cox*1 gene and mtDNA genomes were performed using ClustalW2 in CLC Main Workbench. *Cox*1 sequences of the 23 *R. microplus* ticks were collapsed into haplotypes, using DnaSP v5.10.01 [29]. Percent identity analyses was performed using Clustal Omega multiple sequence analyses tool [30] (https://www.ebi.ac.uk/Tools/msa/clustalo/). A *cox*1 phylogenetic tree was constructed by employing the Maximum Likelihood (ML) algorithm implemented in MEGA X [31] using a total of 42 nucleotide sequences which included three Kwale *R. microplus cox*1 haplotypes and one *R. appendiculatus* sequence, 11 sequences from reference ticks and 28 GenBank reference sequences. The best nucleotide substitution model which gave the lowest Bayesian Information Criterion (BIC) score (3833.705) was Tamura 3-parameter (T92+I) as determined using MEGA X. The rate variation model allowed for some sites to be evolutionarily invariable ([+I], 69.54% sites). All positions with less than 95% site coverage were eliminated (partial deletion option). Clade support was assessed *via* 1000 bootstrap replications. Bootstrap values below 70% were collapsed. There were a total of 403 positions in the final *cox*1 dataset. A mtDNA phylogenetic tree was constructed using complete mtDNA genome sequences of two *R. microplus*, one *R. decoloratus* and one *R. appendiculatus* from this study and 13 reference tick mtDNA sequences available in GenBank. The best substitution model for the mtDNA tree which gave the lowest BIC score (148759.37) was General Time Reversible (GTR) [32]. A discrete Gamma distribution was used to model evolutionary rate differences among sites (5 categories (+G, parameter = 0.5523)). The rate variation model allowed for some sites to be evolutionarily invariable ([+I], 30.59% sites). The analysis involved 17 nucleotide sequences. There were a total of 18,435 positions in the final mtDNA dataset. Evolutionary analyses were conducted in MEGA X.

## Results

### Morphological identification of tick species

**Fig. 2:**
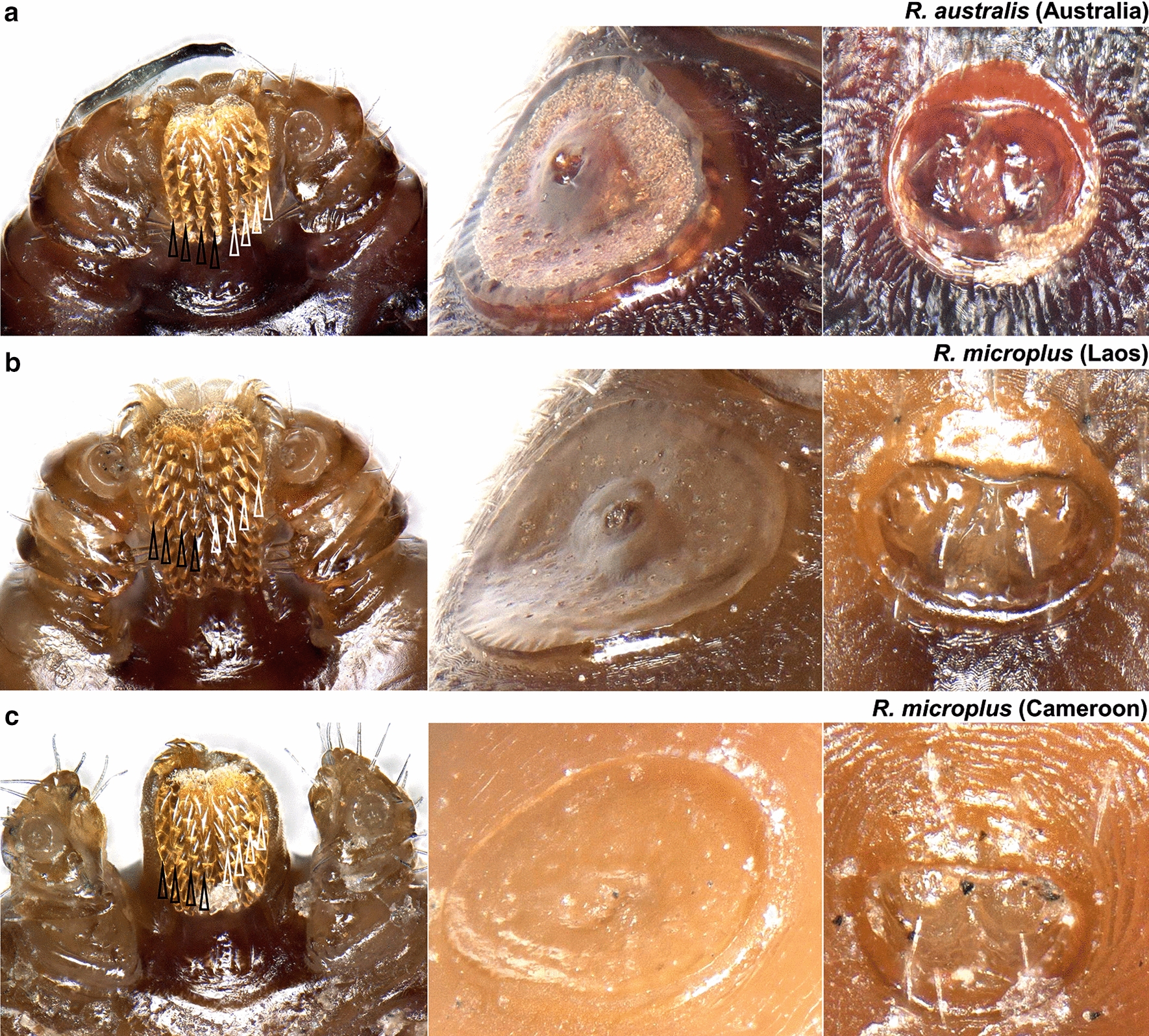
Images of three key morphological features of reference *R. australis* (**a**) and *R. microplus* (**b**, **c**). Left: mouth parts. The characteristic 4 + 4 rows of dentition are shown by black (left row) and clear (right) arrows. Centre: spiracular plate. Right: anal aperture. The three specimens are: **a** *R. australis* (Australia); **b** *R. microplus* (Laos); and **c** *R. microplus* (Cameroon)

**Fig. 3:**
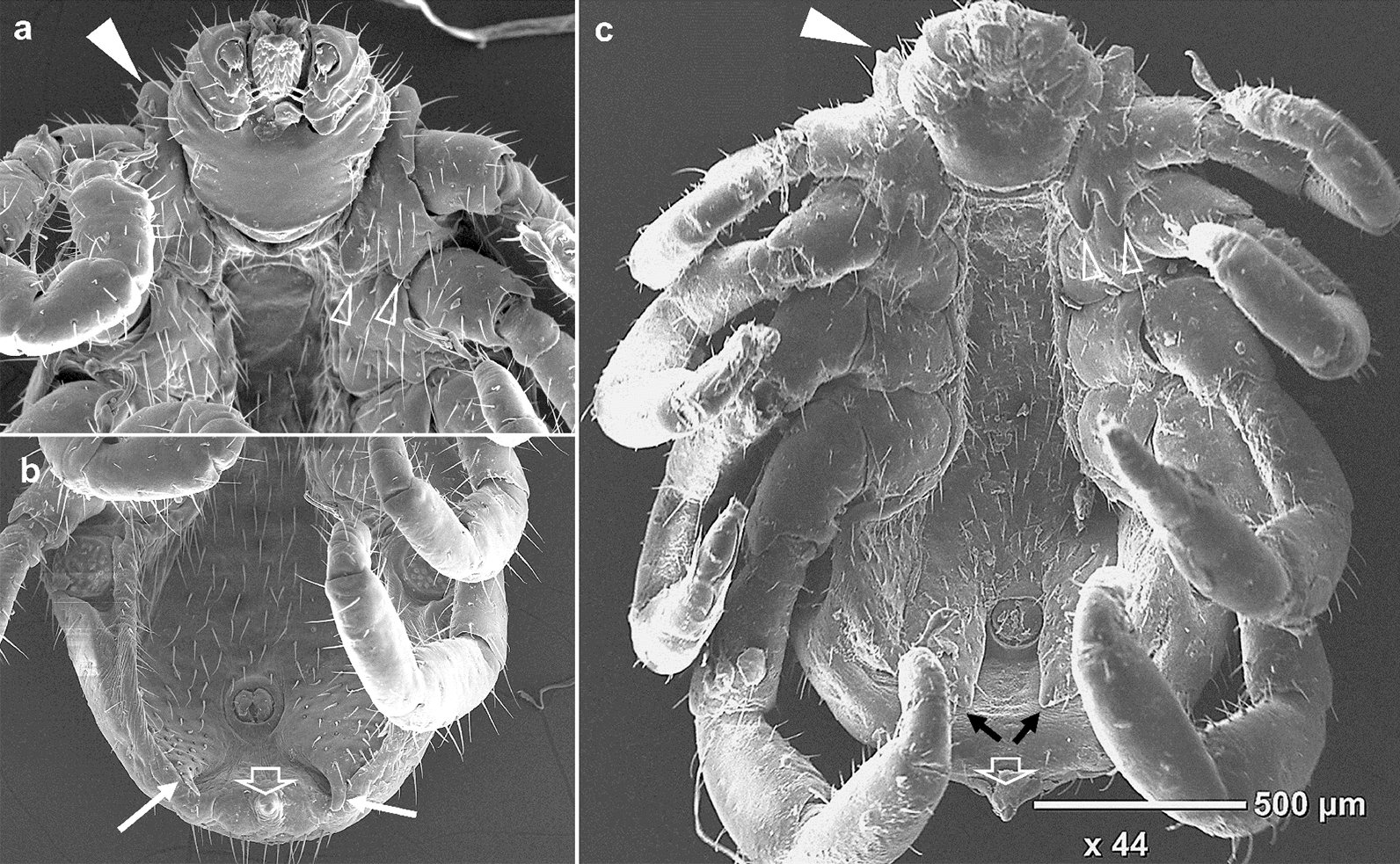
SEM images (ventral view) of male reference *R. decoloratus* and *R. australis* showing some characteristic morphological features **a** Upper anterior body of *R. decoloratus* (Kenya). Block pointed arrow shows coxa I anterior spur. Clear arrows show coxa I posterior spurs which are usually distinct and short in males. **b** Posterior body of *R. decoloratus.* White arrows show long distinct adenal plate spurs that are normally visible dorsally. The clear white arrow shows the caudal appendage which is long and visible dorsally. **c** Complete body of *R. australis* (Australia). Block pointed arrow shows coxa I anterior spur which is usually long and visible dorsally. Clear arrows show coxa I posterior spurs which are distinct and long in males. Black arrows show short indistinct adenal plate spurs which are not visible dorsally. The clear white arrow shows the caudal appendage which is usually short and narrow

**Fig. 4:**
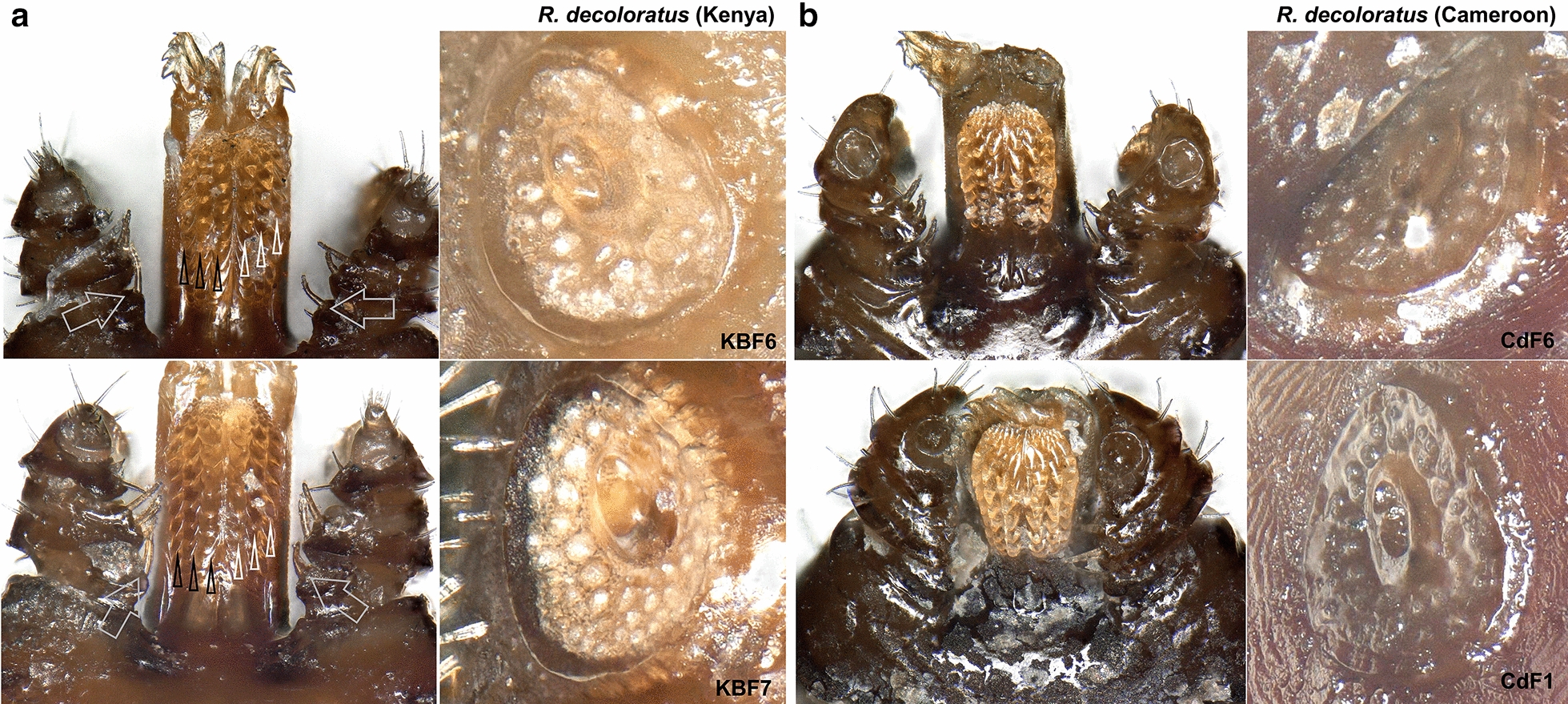
Images of key morphological features of reference *R. decoloratus* from Kenya (**a**) and Cameroon (**b**). Left: mouth parts and spiracular plate images of Kenyan specimens KBF6 (upper plate) and KBF7 (lower plate). Big clear arrows show the characteristic pectinate setae on palp article I. Black and clear-pointed arrows show the characteristic 3 + 3 dentition rows. Right: mouth parts and spiracular plate images of Cameroonian specimens CdF6 (upper plate) and CdF1 (lower plate)

**Fig. 5:**
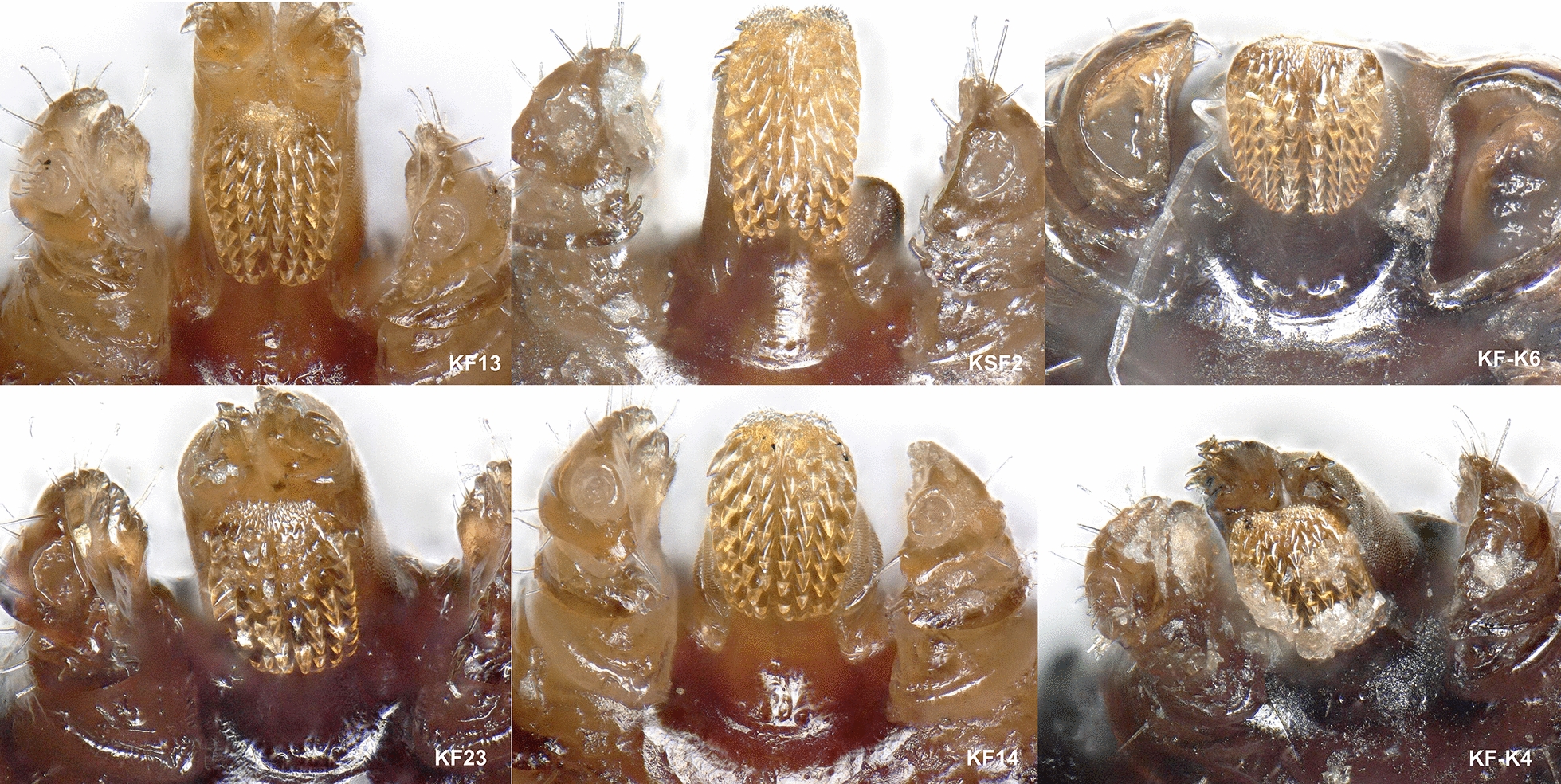
Mouth parts images of six female *R. microplus* specimens from Kwale County. Upper plate: Left: KF13; Middle: KSF2; Right: KFK6. Lower plate: Left: KF23; Middle: KF14; Right: KFK4. Most of the ticks were semi- or fully engorged

Of the 35 ticks collected from Kwale County, Kenya, 16 specimens were semi-engorged, 11 were fully engorged, 3 were partially fed and 5 were slightly fed (Additional file 1: Table S1). Morphologically, all but two ticks belonged to the genus *Rhipicephalus* (*n = *33). Two ticks represented *Amblyomma variegatum.* On microscopic examination, 12 *Rhipicephalus* specimens were found to have damaged or missing mouth parts while 21 specimens were intact. Further examination revealed presence of 10 *R. appendiculatus* specimens. The remaining 23 specimens were compared to reference *R. microplus*, *R. australis* and *R. decoloratus*. Female reference *R. microplus* and *R. australis* had four rows of denticles on each side of the hypostome and a concavity with no setae on the medial aspect of article one of the palpal segment (Fig. 2). Reference *R. decoloratus* had three rows of denticles on each side of the hypostome, females had a convex protuberance with setae on the first palpal segments while males had long distinct adanal plates with long spurs which extended beyond the posterior body margin and were clearly visible outside the scutum (Figs. 3, 4). Twelve ticks were found to have morphological features of female *R. microplus* (Fig. 5). The remaining ticks (*n = *11) had damaged mouth parts but spiracular plate consistent with that of subgenus *Boophilus* (Figs. 2, 3, 4).

### Molecular identification and genetic characterization based on *cox*1

Molecular analysis involving partial amplification and sequencing of the *cox*1 gene was performed for 32 ticks from Kwale, Kenya (12/12 *R. microplus*; 11/11 *Rhipicephalus* sp.; 8/10 *R. appendiculatus*; 1/2 *A. variegatum*). In addition, *cox*1 was generated for 11 reference tick specimens of *R. australis* (Australia, *n* = 3), *R. microplus* (Laos, *n = *2), *R. microplus* (Cameroon, *n* = 2), *R. decoloratus* (Cameroon, *n* = 2) and *R. decoloratus* (Kenya, *n* = 2).

The *cox*1 DNA sequence of all *R. microplus* (*n* = 12) and *Rhipicephalus* sp. (*n* = 11) was > 99.5% identical to each other representing *R. microplus*. Presence of *R. microplus* was demonstrated in all sites where ticks were collected; Kwale Kidimu (6/6), Matuga Tangini (3/6) and Shimoni Kidimu (4/5), as well in across the unspecified location in Kwale County (10/18). Only two informative polymorphic sites were found, resulting in three haplotype sequences differing in two variable polymorphic sites at position 51 and 483 with a haplotype diversity (Hd) of 0.5692. Haplotype 1 consisted of six sequences which had the bases TG while 14 sequences formed haplotype 2 with bases CG at the two variable positions. Haplotype 3 consisted of 3 sequences with bases CA at the two polymorphic sites. The distribution of the three haplotypes across the sampled sites is shown in Additional file 2: Table S2. The *cox*1 sequences of the 23 Kwale *R. microplus* and 11 sequences of reference ticks were deposited in the GenBank database under the accession numbers MT181192–MT181227 (Additional file 1: Table S1), while the three haplotype sequences were deposited under the accession numbers MT181228–MT181230. Three *R. microplus* haplotypes from Kenya were > 99% identical to each of the 10 *R. microplus* available sequences from Africa; Cameroon (CF4, CF5, MK648412, MG983832, MG983831), Democratic Republic of Congo (MF45873), South Africa (KY678117), Benin (KY678120), Madagascar (KY678118) as well as a *cox*1 sequence from a tick collected on a zebra from Kenya (KX228549) (Additional file 3: Table S3). The Kwale *R. microplus cox*1 sequences had an identity of 94.5% to *R. australis* sequences. The *cox*1 sequences from reference *R. microplus* specimens from Laos and GenBank reference sequences from China matched with a lower identity of 92%.

### *cox*1 phylogeny and genetic relationships

**Fig. 6:**
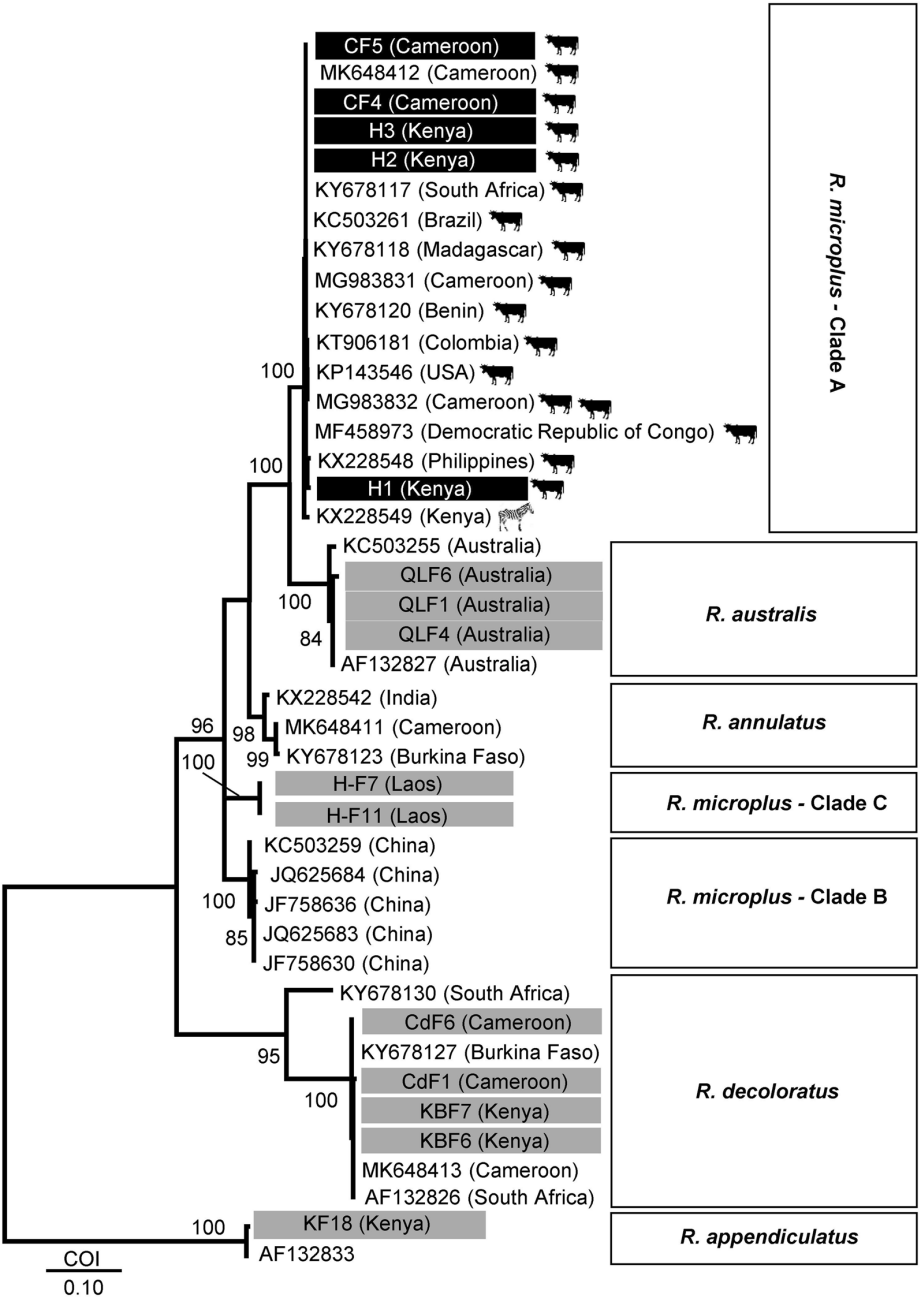
Maximum Likelihood (ML) *cox*1 tree showing the phylogenetic relationships between ticks analysed in this study and 28 reference sequences. The tree was constructed based on Tamura 3-parameter (T92) model [51]. The rate variation model allowed for some sites to be evolutionarily invariable ([+I], 69.54% sites). This analysis involved 42 nucleotide sequences. Tick samples analysed in this study are highlighted. The scale represents 0.10 nucleotide substitutions per site. There was a total of 403 positions in the final dataset. Evolutionary analyses were conducted in MEGA X [31]. Bootstrap values (1000 replications) above 70% are shown

Phylogenetic analysis based on *cox*1 sequences was undertaken to determine the genetic relationships between the Kwale *R. microplus* haplotype sequences and reference *R. microplus*, *R. australis* and *R. decoloratus* (Fig. 6). The Kenyan *R. microplus* clustered in a major clade (*R. microplus* Clade A) together with sequences from Cameroon (CF4 and CF5). The cluster was strongly separated (100%) from a GenBank *R. microplus* sequence from Kenya (KX228549). Clade A was strongly separated (92%) from a *R. australis* sister clade. Two sequences of *R. microplus* specimens from Laos analysed in this study clustered with GenBank reference sequences from China (*R. microplus* Clade B). The Kenyan *R. decoloratus* sequences analysed in this study (KBF6, KBF7) clustered closely in one clade with those from Cameroon (CdF1 and CdF6).

### Mitochondrial genomes characterization and phylogeny

**Fig. 7:**
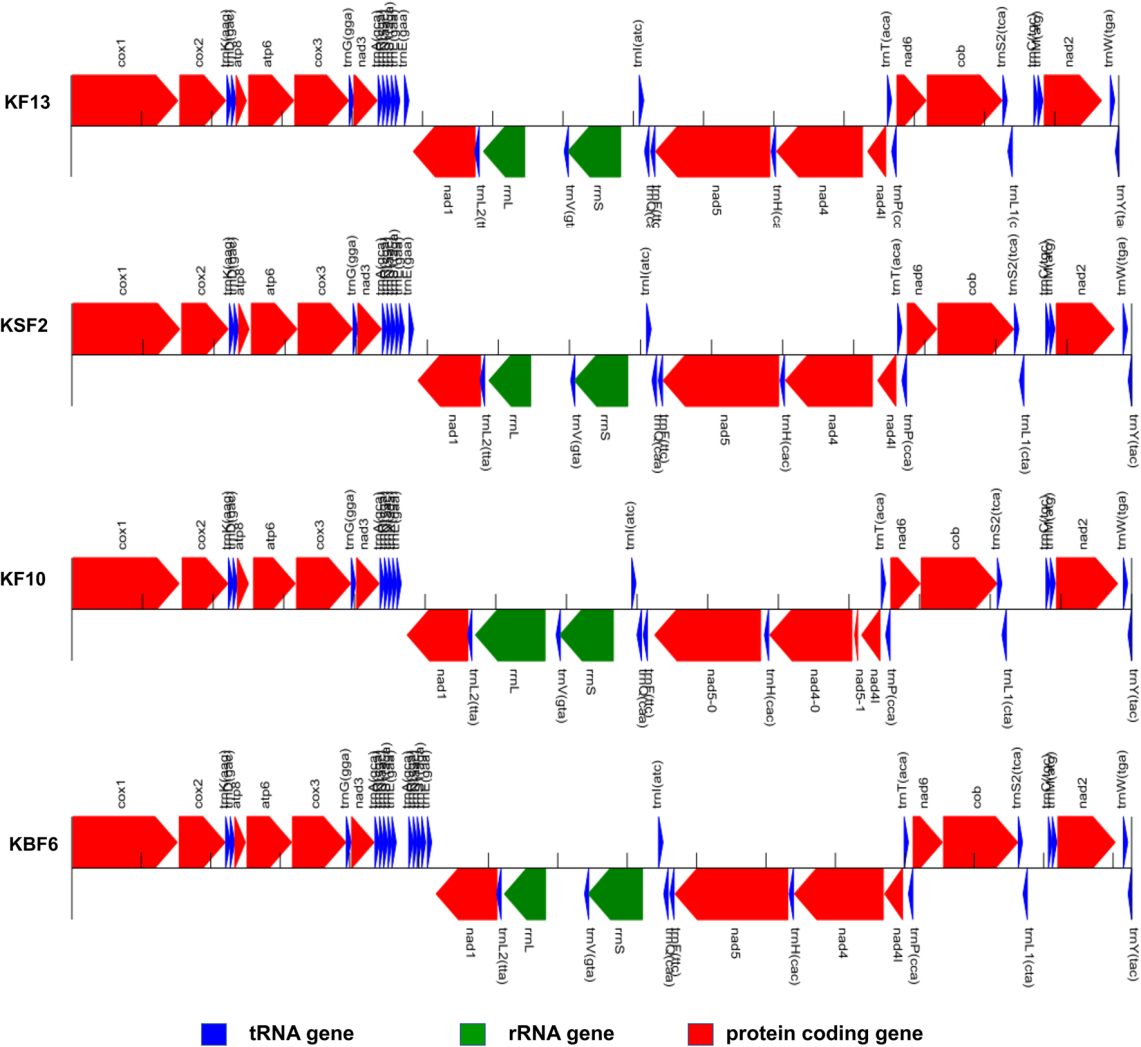
Graphical overview of the 13 mitochondrial proteins, 22 tRNAs and 2 rRNAs annotated by MITOS in the four mitochondrial genomes sequenced in this study. Genes located on the plus strand are drawn in the upper part. Genes annotated on the minus strand are shown in the lower region. A small vertical line is drawn every 1000 nucleotides. KF13 and KSF2 refer to mtDNA genomes of the two *R. microplus* specimens sequenced while KF10 and KBF6 are genomes of *R. appendiculatus* and *R. decoloratus* respectively

Four new complete mitochondrial genomes (mtDNA) were assembled from Illumina reads from two specimens of *R. microplus* from Kwale (KF13, KSF2), one *R. apendiculatus* from Kwale (KF10) and from a specimen representing *R. decoloratus* colony at ILRI Kenya (KBF6) (MT430985–MT430988; Additional file 1: Table S1). MITOS, a web server for automatic annotation of metazoan mtDNA was used to annotate protein coding genes, tRNAs and non-coding RNAs in the four complete mtDNA genomes. Multiple sequence (MS) analysis was performed on the four mtDNA sequences and 13 reference genomes from GenBank. These included six *R. microplus*, one *R. annulatus*, one unverified *R. decoloratus*, one *R. geigyi*, a partial *R. appendiculatus*, one *R. sanguineus*, one *R. turanicus* and one *H. longicornis* reference genome sequences. There were 13 protein-coding genes, 22 tRNAs and two rRNAs annotated (Fig. 7).

From the MITOS prediction, the arrangement and length of the annotated mitochondrial features in the two *R. microplus* (KF13 and KSF2) genomes was very similar (Fig. 7). The program predicted the presence of two trnE genes (65 bp) in the two genomes. The first occurred in the plus strand in position 4614–4678 while the second was in position 4744–4808 for both KF13 and KSF2. Both predictions had a similar e-value of 3.313e × 10^−5^. In the MS alignment, this region was part of control region I which occurs within the tandem repeat region annotated between *trnE* and *nad*1 in other reference *R. microplus* genomes. The program also predicted pseudo-copies of tRNA genes for *trnA* (gca), *trnR* (cga), *trnN* (aac), *trnS1*(aga) and *trnE* (gaa) (Fig. 7) in position 4854–5184 of the *R. decoloratus* (KBF6) mt genome. The MS alignment showed the first prediction in position 4368–4610 to be conserved and common to the reference mtDNAs. The pseudo-prediction from position 4854–5184 was a 331 bp long AT rich sequence lacking in the unverified *R. decoloratus* reference sequence and in other reference genomes. It occurs as part of the tandem repeat region annotated between *trnE* and *nad*1 in other reference *R. microplus* genomes. MITOS could not locate the gene for *trnS*1 in the *R. apendiculatus* (KF10) genome. In the reference mtDNA annotations, the *trnS*1 gene is reasonably conserved located upstream of the *trnN* gene in position 4557–4612 for KF13 and KSF2 and 4555–4610 for KBF6. In the *R. appendiculatus* KF10 sequence, the region (4543–4599) is predicted by MITOS to be a pseudo non-standard tRNA feature (trnX) (Fig. 7). In the MS alignment, two nucleotides are missing from the 5′-start of the gene in KF10 and the partial *R. appendiculatus* Zimbabwe reference genome when compared to the predicted *trnS*1 gene of the *R. microplus* reference genomes. MITOS also predicted a short *nad*4 gene and two *nad*5 genes in the minus strand in KF10 (Fig. 7). The first *nad*5 (1500 bp long) at position 8251–9750 with a quality value of 2.05 × 10^8^ and a second tiny pseudo-fragment (45 bp) at position 11081–11125 with a value of 375.9. With such a lower value, the second prediction is highly unlikely. In the MS alignment, this fragment is part of *nad*4 which appears to be conserved across the other reference genomes.

**Fig. 8:**
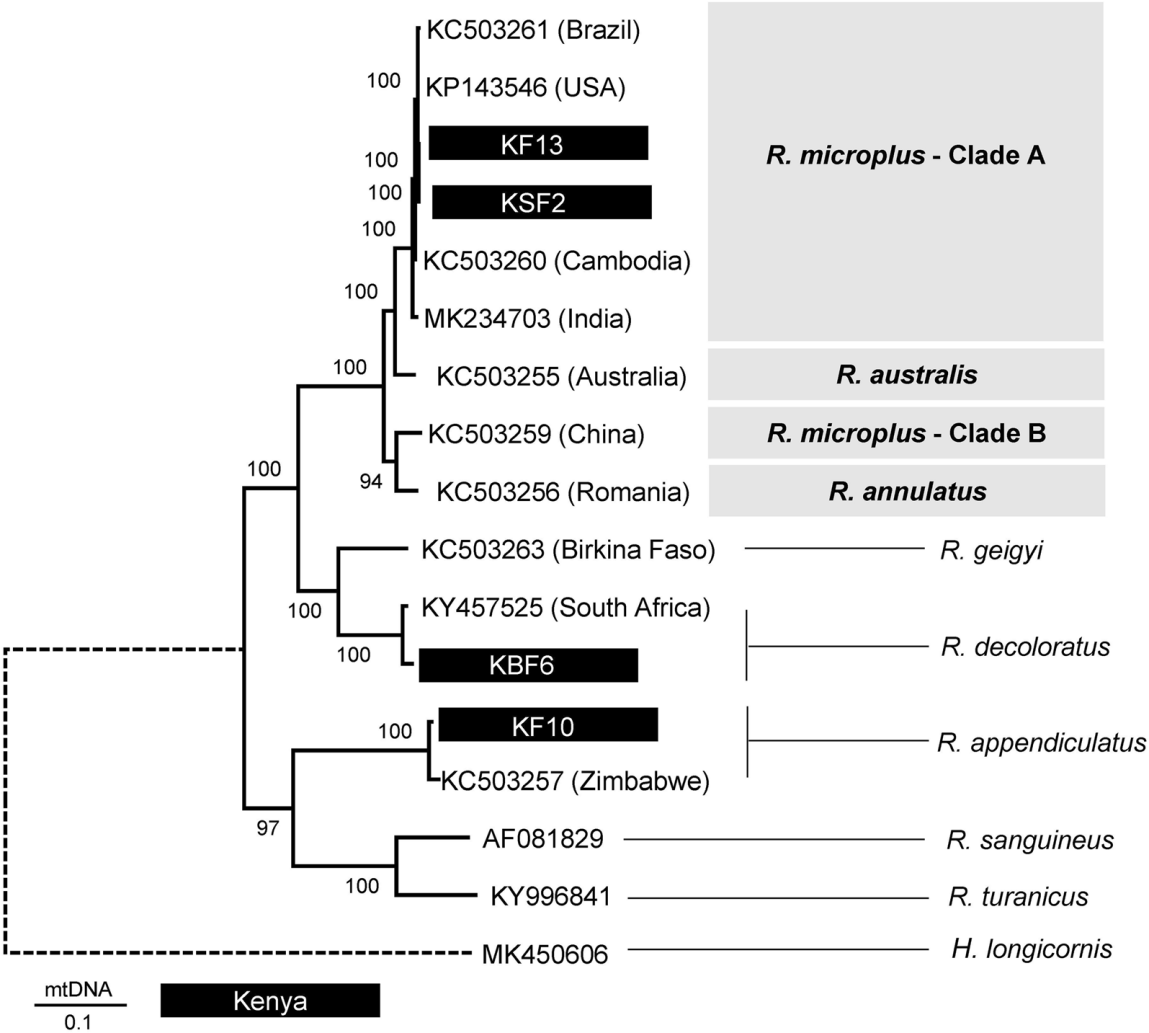
Maximum Likelihood (ML) mtDNA tree inferred from 17 nucleotide sequences. The analysed sequences included four mitochondrial genomes sequenced in this study (highlighted) and annotated reference genomes available on GenBank. The four mtDNA genomes analysed in this study were: *R. microplus* KF13 and KSF2, *R. appendiculatus* KF10 and *R. decoloratus* KBF6. Six *R. microplus* genomes and one genome each for *R. annulatus*, *R. geigyi*, *R. sanguineus*, *R. turanicus* and *H. longicornis* from GenBank were included. A partial mtDNA genome of *R. appendiculatus* (KC503257) and an unverified *R. decoloratus* genome (KY457525) available on GenBank were used to compare the genome sequences of *R. appendiculatus* and *R. decoloratus*. The tree was reconstructed using the General Time Reversible (GTR) (Nei & Kumar [32]) in MEGA X. There was a total of 18,435 positions in the final dataset

The two *R. microplus* mtDNA (KF13 and KSF2) had high nucleotide similarity (> 99%) and matched with an identity of greater than > 98% to the *R. microplus* reference genome sequences from India, Cambodia, Brazil and Texas (USA) (Additional file 4: Table S4). They matched to the *R. australis* reference genome (KC503255) with a similarity of 96% and to the China sequence (KC503259) with an identity of 94%. Their similarity to the *R. decoloratus* sequence (KBF6) was 87% and 83% to the *R. appendiculatus* sequence (KF10). In the mtDNA phylogenetic tree, four major clusters were observed with all the nodes strongly supported by 100% bootstrap value (Fig. 8). The two *R. microplus* genome sequences (KF13 and KSF2) clustered closely with *R. microplus* reference sequences from Brazil (KC503261), USA (KP143546), Cambodia (KC503260) and India (MK234703).

### Molecular detection of *B. bovis* in *R. microplus* ticks

Genomic DNA samples from 23 specimens confirmed by molecular analysis to be *R. microplus* were subjected to sensitive molecular quantitative qPCR assays to detect presence of bovine blood and consequently *B. bovis* parasites. Presence of cattle blood DNA was tested in undiluted (neat) DNA by amplifying mammalian glyceraldehyde 3-phosphate dehydrogenase (GAPDH). The cycle quantification (Cq) values ranged from a high of 39.45 in KSF3 to a low of 23.40 in KF16. Bovine DNA was not detected in 2 samples (KF-K6 and KSF5), analysis of a 1:10 diluted DNA of these two samples resulted in the detection of bovine DNA (Cq value of 37.26) in KSF5, but no DNA was detected in the diluted KF-K6 sample.

Having confirmed the presence of bovine DNA in the extracted tick DNA, *B. bovis-*specific primers were used to amplify specific regions of cytochrome *b* (*cytb*) and *18S* rDNA genes to detect presence of *B. bovis* parasites. At a threshold of 100 RFU, all material was considered negative for *B. bovis* DNA using either assay (Cq values ≥ 40.00).

## Discussion

The invasive *R. microplus*, a highly efficient vector of tick fevers caused by *B. bovis*, *B. bigemina* and *A. marginale* has rapidly spread into many African countries over the last decade [3–11]. In many areas, it has been reported to progressively displace the indigenous *R. decoloratus* and *R. annulatus* [3, 5, 11, 33–36] sometimes establishing itself in areas predicted to be climatically unsuitable for its survival [7, 33, 36]. The latest findings of widespread occurrence of *R. microplus* in eastern Uganda [11] and recent introduction and spread of the tick in Cameroon [10] have been attributed to uncontrolled live animal movements from neighbouring countries and cross-border animal trade. To date, the widely accepted genetic characterizations and phylogenetic clustering of *R. microplus* are based on the *cox*1 gene. Therefore, we applied the same marker to characterize and genotype Kenyan *Boophilus* ticks.

In Kenya, *R. microplus* was first recorded in 1974 in a limited area along the Kenyan coast [16]. This study confirms that *R. microplus* still occurs in the region, although *B. bovis* was not detected in the limited number of ticks analysed. The coastal plain in Kwale is usually hot and humid with an average temperature above 25 °C and precipitation greater than 1000 mm annually [37]. In southern Africa, *R. microplus* has been shown to prefer warm and humid conditions [38]. Warm temperatures of at least 15–20 °C are required for egg-laying and larval hatching to occur. Thus, the eco-climatic conditions in Kwale are suitable for the survival of *R. microplus*. The tick is widespread over most of the neighbouring Tanzanian mainland [36]. The cross-border animal market at Tanga and transhumance at Shimoni port could be contributing to the re-introduction and spread of *R. microplus* from mainland Tanzania and the Indian ocean Islands into Kenya.

The *cox*1 gene has previously been shown to have greater intraspecies resolution within the *R. microplus* complex compared to *12S*, *16S* genes or ITS2 region [20, 21, 39, 40]. Based on *cox*1 phylogenetics, five distinct generic clusters have been shown to occur within the *R. microplus* species complex [20, 21, 40]. These include Clade A (ticks from Africa, Asia and South America) and Clade B (southern China and northern India) of Burger et al. [20]; Clade C (ticks from Malaysia and India) of Low et al. [21]. *Rhipicephalus australis* and *R. annulatus* have been found to be closely related to ticks in Clade B *sensu* Burger et al. [20]. The Kenyan *R. microplus* sequences analysed in this study clustered with other African *R. microplus* reference sequences into Clade A *sensu* Burger et al. [20].

Previous studies on genetic diversity and population structure of *R. microplus* have reported a low genetic variation and differentiation. Low et al. [21] reported a relatively low but significant genetic differentiation of *R. microplus* in Malaysia while in South African *R. microplus* a low variation within species was observed with *cox*1 and *16S* rDNA genes [39]. A similar low divergence was also recently observed in Cameroonian *R. microplus* ticks by Silatsa et al. [10] using the two mitochondrial genes. Low levels of genetic differentiation and lack of population structuring amongst *R. microplus* populations based on microsatellite markers has also been observed [39, 41, 42]. In this study, we found a low divergence and lack of geographical sub-structuring among the African *cox*1 sequences analysed. The same low divergence has been observed with the *16S* rRNA gene [10, 21].

A one-host tick, *R. microplus*, completes its entire parasitic life-cycle on a single host and is considered a cattle specialist [43]. Cattle are thought to be the only effective maintenance vertebrate hosts of *R. microplus* and although it can infest sheep, goats and other wild bovids, infestations of other hosts only occur when a population of ticks is maintained on cattle [22]. De Meeûs et al. [42] reported the divergence of *R. microplus* into two differentiated host-specific genetic groups with little or no genetic exchange within a short period of time after the tick was introduced in New Caledonia. One group was specific to cattle and another specific to the rusa deer which often co-grazes with cattle. This strict host specialization and the observed sympatric isolation can limit genetic exchange and flow resulting in very low genetic variation. All *cox*1 sequences analysed in this study were derived from ticks collected from cattle except for one Kenyan sequence (GenBank: KX228549) that had been collected from a migratory zebra [44]. Although *R. microplus* has been found to infest other livestock and some African wild ungulates including the African Cape buffalo, the role of African wildlife as reservoirs of *R. microplus* is currently not clear. A genetic drift caused by tick population bottlenecks such as a small effective population size or a founder event where a new population arises from few individuals such as was observed in New Caledonia can result in a decrease in allele frequencies leading to the low genetic variation and differentiation observed with *R. microplus* [42]. Analysis of a larger set of ticks from geographically separated areas in Africa using a combination of markers may reveal the gene flow patterns and population structure of *R. microplus* as it adapts into new areas.

Although *R. microplus* was first recorded in Kenya in the mid-1970s, it had been reported in Tanzania in the late 1960s [45]. Though the tick is thought to have been introduced into East and South Africa through cattle importations from Asia following the rinderpest epidemic in 1896 [2], the occurrences in eastern Africa were attributed to the documented spread of the tick from South Africa to the rest of southern and eastern African regions [22, 36]. Despite the different historical occurrences of *R. microplus* in Asia, Africa and South America, we observed the grouping of *R. microplus* from Africa, Asia and South America into the same cluster (Clade A). Although the use of markers with low discrimination power may contribute to the low genetic variation observed, the *cox*1 gene used has previously been shown to have high intraspecies resolution within the *R. microplus* complex resulting in the formation of the five known genetic groups [20, 21, 40]. Genetic variation within ticks has been shown to be influenced by several factors including the mammalian host range (specialist *vs* generalist), eco-climatic factors as well as geographical barriers to gene flow [43, 46, 47]. Although the numbers analysed in this study are few and from one general area, when combined with available data on other African genotypes, the sequence identities and *cox*1 clustering observed suggest a lack of phylogeographic structuring of *R. microplus* in Africa. It was anticipated that isolation-by-distance would result in the eastern Africa ticks being genetically divergent from those from Central and West Africa. However, based on *cox*1, our findings reveal that there is very low divergence of *R. microplus* populations from East, South, West and Central Africa.

Analysis of whole mitochondrial (mt) genomes of two Kenyan *R. microplus* confirmed the high conservation of mtDNA genome within the *R. microplus* complex as observed by Burger et al. [20]. The arrangement and length of the 13 mitochondrial proteins, 22 tRNAs (trn) and two rRNAs (rrn) in the two Kwale *R. microplus* genomes is similar to the reference genomes reported by Burger et al. [20]. The pseudo-*trnE* gene annotated by MITOS in the two Kenyan *R. microplus* genomes appears to be part of the tandem repeat region found to be present in all members of the *R. microplus* complex [20, 48]. This 150-bp tandem repeat consists of the 3′-end of *trnS*, *trnE*, the Tick-Box motif and the 3′-end of *nad*1. The Tick-Box is a 17-bp motif which intersperses the 3′-ends of *nad*1 and *16S* rRNA during transcription in tick mt genomes [49]. Pseudo-copies of tRNA genes for *trnA* (gca), *trnR* (cga), *trnN* (aac), *trnS*1(aga) and *trnE* (gaa) were predicted in *R. decoloratus* (KBF6) mt genome (Fig. 7). This 331-bp insert occurred within the variable tandem repeat region annotated in other *R. microplus* reference genomes. It is lacking in the unverified *R. decoloratus* reference sequence and in other reference genomes. Two nucleotides were found to be missing from the 5′-start of the *trnS*1 gene in the annotated *R. appendiculatus* mtDNA and the partial *R. appendiculatus* Zimbabwe reference genome when compared to the predicted *trnS*1 gene of *R. microplus* reference genomes.

In Kenya and in many other African countries, diagnosis of tick-borne diseases including babesiosis is mostly achieved through observation of clinical signs and microscopic examination of blood smears, methods which are rapid and inexpensive but not specific or sensitive especially in immune carrier animals. The use of sensitive and specific molecular assays may increase and improve detection of tick-borne hemoparasites as indicated by the detection of *B. bovis* in Kenya [18, 19]. Highly sensitive quantitative PCR (qPCR) assays based on SYBR green [50] and TaqMan probes [26, 27] able to detect, quantify and differentiate *B. bovis* from *B. bigemina* are available. Validated sensitive diagnostic and epidemiological assays should be employed to screen and confirm the status and distribution of *B. bovis* in Kenya and other Africa countries where *R. microplus* has recently spread.

## Conclusions

Our study has confirmed the occurrence of *R. microplus* in Kenya. The Kenyan *R. microplus cox*1 sequences clustered very closely with reference African genotypes. We found very low levels of genetic variation and lack of geographical sub-structuring among the African *cox*1 sequences belonging to *R. microplus* Clade A. With current invasion and rapid spread of *R. microplus* in many African countries, it is important to determine whether its occurrence is accompanied by the presence of the pathogenic *B. bovis*. Our findings and the recent reports of widespread occurrence of *R. microplus* in Africa provide the fundamental basis and rationale to implement diagnosis, urgent strategic control and surveillance to determine and monitor the spread of *R. microplus* and *B. bovis* in Kenya and in the region.

## Supplementary information

**Additional file 1: Table S1.** Detailed descriptions of the ticks analysed in the current study.

**Additional file 2: Table S2.** Distribution of three *cox*1 haplotype sequences obtained in this study across sampled sites.

**Additional file 3: Table S3.** *cox*1 sequences percent (%) identity matrix.

**Additional file 4: Table S4.** Mitochondrial DNA (mtDNA) sequences percent (%) identity matrix.

## Data Availability

The nucleotide dataset(s) supporting the conclusions of this article are available in the GenBank repository (http: //www.ncbi.nlm.nih.gov/genbank/). The 23 Kenyan *R. microplus*, haplotype and reference ticks *cox*1 sequences are under the accession numbers MT181192–MT181230. The whole mitochondrial genome DNA datasets appear in GenBank under the accession numbers MT430985–MT430988. The whole genome sequence data are available at SRA NCBI BioProject: PRJNA611067 (https://www.ncbi.nlm.nih.gov/bioproject/PRJNA611067). The exoskeletons of voucher specimens of the 35 Kenyan and 11 reference ticks characterized in this study have been deposited with the Australian National Insect Collection (ANIC) Museum, Canberra.

## Abbreviations

BLASTN: basic local alignment search tool
BIC: Bayesian information criterion
*cox*1: cytochrome *c* oxidase subunit 1
Cq: cycle quantification
DNA: deoxyribonucleic acid
dNTPs: deoxyribonucleotide triphosphates
ILRI: International Livestock Research Institute
GTR: general time reversible
mt: mitochondrial
mtDNA: mitochondrial DNA
MS: multiple sequence
rDNA: ribosomal deoxyribonucleic acid
qPCR: quantitative PCR
SEM: scanning electron microscopy
trn: transfer RNA
rrn: ribosomal RNA
