# Supplementary material for: Molecular evidence confirms occurrence of Rhipicephalus microplus Clade A in Kenya and sub-Saharan Africa
Source: Parasit Vectors. 2020 Aug 27;13:432. doi: 10.1186/s13071-020-04266-0 (PMC7453536; doi:10.1186/s13071-020-04266-0)
Supplement: Supplementary file 1 — Additional file 1: Table S1. Detailed descriptions of the ticks analysed in the current study. [file 13071_2020_4266_MOESM1_ESM.docx]

**Additional file 1: Table S1. Detailed descriptions of the ticks analysed in the current study**

| **No** | **Sample ID** | **Source/ Locality** | **Sex** | **Life stage** | **Engorgement** | **Voucher state** | **Host** | **Morphology** | **Molecular ID** | **COI GenBank Accession Number** | **mtDNA GenBank Accession Number** |
| --- | --- | --- | --- | --- | --- | --- | --- | --- | --- | --- | --- |
| 1 | KF21 | Matuga Tangini, Kenya | Female | Adult | Engorged | Damaged mouth | Cattle herd | *Rhipicephalus spp.* | *R. microplus* | MT181192 |  |
| 2 | KF7 | Matuga Tangini, Kenya | Female | Adult | Semi-Engorged | Damaged mouth | Cattle herd | *Rhipicephalus spp.* | *R. microplus* | MT181193 |  |
| 3 | KF20 | Matuga Tangini, Kenya | Female | Adult | Engorged | Damaged mouth | Cattle herd | *Rhipicephalus spp.* | *R. microplus* | MT181194 |  |
| 4 | KSF1 | Shimoni Kidimu, Kenya | Female | Adult | Partially fed | Damaged mouth | Cattle herd | *Rhipicephalus spp.* | *R. microplus* | MT181195 |  |
| 5 | KF-K6 | Kwale Kidimu, Kenya | Female | Adult | Semi-Engorged | Intact | Cattle herd | *R. microplus* | *R. microplus* | MT181196 |  |
| 6 | KF23 | Kwale, Kenya | Female | Adult | Engorged | Intact | Cattle herd | *R. microplus* | *R. microplus* | MT181197 |  |
| 7 | KSF2 | Shimoni Kidimu, Kenya | Female | Adult | Partially fed | Intact | Cattle herd | *R. microplus* | *R. microplus* | MT181198 | MT430986 |
| 8 | KF4 | Kwale, Kenya | Female | Adult | Engorged | Damaged mouth | Cattle herd | *Rhipicephalus spp.* | *R. microplus* | MT181199 |  |
| 9 | KF-K1 | Kwale Kidimu, Kenya | Female | Adult | Engorged | Intact | Cattle herd | *R. microplus* | *R. microplus* | MT181200 |  |
| 10 | KSF3 | Shimoni Kidimu, Kenya | Female | Adult | Partially fed | Damaged mouth | Cattle herd | *Rhipicephalus spp.* | *R. microplus* | MT181201 |  |
| 11 | KF-K5 | Kwale Kidimu, Kenya | Female | Adult | Engorged | Damaged mouth | Cattle herd | *Rhipicephalus spp.* | *R. microplus* | MT181202 |  |
| 12 | KF-K2 | Kwale Kidimu, Kenya | Female | Adult | Engorged | Damaged mouth | Cattle herd | *Rhipicephalus spp.* | *R. microplus* | MT181203 |  |
| 13 | KF13 | Kwale, Kenya | Female | Adult | Semi-Engorged | Intact | Cattle herd | *R. microplus* | *R. microplus* | MT181204 | MT430985 |
| 14 | KF1 | Kwale, Kenya | Female | Adult | Semi-Engorged | Damaged mouth | Cattle herd | *R. microplus* | *R. microplus* | MT181205 |  |
| 15 | KF-K3 | Kwale Kidimu, Kenya | Female | Adult | Engorged | Intact | Cattle herd | *R. microplus* | *R. microplus* | MT181206 |  |
| 16 | KF24 | Kwale, Kenya | Female | Adult | Semi-Engorged | Intact | Cattle herd | *R. microplus* | *R. microplus* | MT181207 |  |
| 17 | KF16 | Kwale, Kenya | Female | Adult | Semi-Engorged | Damaged mouth | Cattle herd | *Rhipicephalus spp.* | *R. microplus* | MT181208 |  |
| 18 | KF5 | Kwale, Kenya | Female | Adult | Semi-Engorged | Damaged mouth | Cattle herd | *Rhipicephalus spp.* | *R. microplus* | MT181209 |  |
| 19 | KSF5 | Shimoni Kidimu, Kenya | Female | Adult | Semi-Engorged | Intact | Cattle herd | *R. microplus* | *R. microplus* | MT181210 |  |
| 20 | KF2 | Kwale, Kenya | Female | Adult | Engorged | Damaged mouth | Cattle herd | *Rhipicephalus spp.* | *R. microplus* | MT181211 |  |
| 21 | KF14 | Kwale, Kenya | Female | Adult | Semi-Engorged | Intact | Cattle herd | *R. microplus* | *R. microplus* | MT181212 |  |
| 22 | KF11 | Kwale, Kenya | Female | Adult | Semi-Engorged | Intact | Cattle herd | *R. microplus* | *R. microplus* | MT181213 |  |
| 23 | KF-K4 | Kwale Kidimu, Kenya | Female | Adult | Engorged | Intact | Cattle herd | *R. microplus* | *R. microplus* | MT181214 |  |
| 24 | CF4 | Cameroon | Female | Adult | partially fed | Entire | Cattle herd | *R. microplus* | *R. microplus* | MT181215 |  |
| 25 | CF5 | Cameroon | Female | Adult | partially fed | Entire | Cattle herd | *R. microplus* | *R. microplus* | MT181216 |  |
| 26 | QLF1 | Australia | Female | Adult | Unfed | Entire | Lab colony | *R. australis* | *R. australis* | MT181217 |  |
| 27 | QLF4 | Australia | Female | Adult | Unfed | Entire | Lab colony | *R. australis* | *R. australis* | MT181218 |  |
| 28 | QLF6 | Australia | Female | Adult | Engorged | Entire | Lab colony | *R. australis* | *R. australis* | MT181219 |  |
| 29 | H-F7 | Laos | Female | Adult | Unfed | Entire | Cattle herd | *R. microplus* | *R. microplus* | MT181220 |  |
| 30 | H-F11 | Laos | Female | Adult | partially fed | Entire | Cattle herd | *R. microplus* | *R. microplus* | MT181221 |  |
| 31 | KBF6 | Kenya | Female | Adult | Unfed | Entire | Lab colony | *R. decoloratus* | *R. decoloratus* | MT181222 | MT430987 |
| 32 | KBF7 | Kenya | Female | Adult | Unfed | Entire | Lab colony | *R. decoloratus* | *R. decoloratus* | MT181223 |  |
| 33 | CdF1 | Cameroon | Female | Adult | semi-Engorged | Entire | Cattle herd | *R. decoloratus* | *R. decoloratus* | MT181224 |  |
| 34 | CdF6 | Cameroon | Female | Adult | semi-Engorged | Entire | Cattle herd | *R. decoloratus* | *R. decoloratus* | MT181225 |  |
| 35 | KM3 | Kwale, Kenya | Male | Adult | slight feed | Intact | Cattle herd | *R. appendiculatus* | *R. appendiculatus* | MT181227 |  |
| 36 | KF18 | Kwale, Kenya | Female | Adult | slight feed | Intact | Cattle herd | *R. appendiculatus* | *R. appendiculatus* | MT181226 |  |
| 40 | KF8 | Matuga Tangini, Kenya | Female | Adult | semi-Engorged | Intact | Cattle herd | *R. appendiculatus* | *R. appendiculatus* | n/a |  |
| 41 | KF22 | Kwale, Kenya | Female | Adult | semi-Engorged | Intact | Cattle herd | *R. appendiculatus* | *R. appendiculatus* | n/a |  |
| 42 | KF15 | Kwale, Kenya | Female | Adult | Engorged | Intact | Cattle herd | *R. appendiculatus* | *R. appendiculatus* | n/a |  |
| 43 | KF12 | Matuga Tangini, Kenya | Female | Adult | Semi-Engorged | Intact | Cattle herd | *R. appendiculatus* | *R. appendiculatus* | n/a |  |
| 44 | KF10 | Kwale, Kenya | Female | Adult | Semi-Engorged | Intact | Cattle herd | *R. appendiculatus* | *R. appendiculatus* | n/a | MT430988 |
| 45 | KF9 | Matuga Tangini, Kenya | Female | Adult | Semi-Engorged | Intact | Cattle herd | *R. appendiculatus* | *R. appendiculatus* | n/a |  |
| 46 | KSF4 | Shimoni Kidimu, Kenya | Female | Adult | semi-Engorged | Damaged mouth | Cattle herd | *A. variegatum* | *A. variegatum* | n/a |  |
| 47 | KM6 | Kwale, Kenya | Male | Adult | slight feed | Intact | Cattle herd | *R. appendiculatus* | *n/a* | n/a |  |
| 48 | KM17 | Kwale, Kenya | Male | Adult | slight feed | Damaged mouth | Cattle herd | *A. variegatum* | *n/a* | n/a |  |
| 49 | KM19 | Kwale, Kenya | Male | Adult | slight feed | Intact | Cattle herd | *R. appendiculatus* | *n/a* | n/a |  |

A total of 49 tick specimens were subjected to morphological analyses. However, only 46 (number 1-46) were subjected to further molecular characterizations. These included 35 ticks from Kwale which comprised of 23 *R. microplus* (highlighted orange), 10 *R. appendiculatus (grey)* and two *A. variegatum* (yellow). Eleven reference ticks that included three *R. australis* from Australia (blue), two *R. microplus* (orange) and two *R. decoloratus* (green) from Cameroon, two specimens of *R. microplus* (orange) from Laos and two of *R. decoloratus* (green) from a laboratory colony maintained at the International Livestock Research Institute, Nairobi were also analysed. Three samples (number 47-49) were not subjected to molecular analysis. *cox*1 sequences of 35 ticks (Number 1-36) were deposited in GenBank samples. Mitochondria genomes were obtained for four specimens, two Kenyan *R. microplus* (KF13 and KSF2), one Kenyan *R. decoloratus* (KBF6) and one *R. appendiculatus* (KF10).
