# Supplementary material for: Molecular evidence confirms occurrence of Rhipicephalus microplus Clade A in Kenya and sub-Saharan Africa
Source: Parasit Vectors. 2020 Aug 27;13:432. doi: 10.1186/s13071-020-04266-0 (PMC7453536; doi:10.1186/s13071-020-04266-0)
Supplement: Supplementary file 2 — Additional file 2: Table S2. Distribution of three cox1 haplotype sequences obtained in this study across sampled sites. [file 13071_2020_4266_MOESM2_ESM.docx]

**Additional file 2: Table S2. Distribution of three *cox*1 haplotype sequences obtained in this study across sampled sites**

| **Sampling Site** | **Specimen** | **Haplotype** | **No. of sequences** |
| --- | --- | --- | --- |
| Kwale | KF1, KF2, KF5, KF11, KF13, KF14, KF16, KF23, KF24, | Hap2 | 9 |
|  | KF4 | Hap3 | 1 |
| Kwale Kidimu | KF-K2, KF-K5 | Hap1 | 2 |
|  | KF-K1, KF-K6 | Hap2 | 2 |
|  | KF-K3, KF-K4 | Hap3 | 2 |
| Matuga Tangini | KF7, KF20, KF21 | Hap1 | 3 |
| Shimoni Kidimu | KSF3 | Hap1 | 1 |
|  | KSF1, KSF2, KSF5 | Hap2 | 3 |

Two variable polymorphic sites at position 51 and 483 of the *cox*1 gene were observed. Haplotype 2 with bases CG at the two variable positions had the most sequences (14) distributed across the sampled sites in Kwale. Haplotype 1 which had bases TG consisted of 6 COI sequences most of which (3/6) were from Matuga Tangini. Haplotype 3 with bases CA at the two polymorphic sites had 3 sequences two of which were from Kwale Kidimu.
