# Supplementary material for: Molecular evidence confirms occurrence of Rhipicephalus microplus Clade A in Kenya and sub-Saharan Africa
Source: Parasit Vectors. 2020 Aug 27;13:432. doi: 10.1186/s13071-020-04266-0 (PMC7453536; doi:10.1186/s13071-020-04266-0)
Supplement: Supplementary file 3 — Additional file 3: Table S3. cox1 sequences percent (%) identity matrix. [file 13071_2020_4266_MOESM3_ESM.docx]

**Additional file 3: Table S3. *cox*1 sequences percent (%) identity matrix**

| **No** | **Sequence** | **1** | **2** | **3** | **4** | **5** | **6** | **7** | **8** | **9** | **10** | **11** | **12** | **13** | **14** | **15** | **16** | **17** | **18** | **19** | **20** | **21** | **22** | **23** | **24** | **25** | **26** | **27** | **28** | **29** | **30** | **31** | **32** | **33** | **34** | **35** | **36** | **37** | **38** | **39** | **40** | **41** | **42** |
| --- | --- | --- | --- | --- | --- | --- | --- | --- | --- | --- | --- | --- | --- | --- | --- | --- | --- | --- | --- | --- | --- | --- | --- | --- | --- | --- | --- | --- | --- | --- | --- | --- | --- | --- | --- | --- | --- | --- | --- | --- | --- | --- | --- |
| **1** | KY678130 | 100 | 91.21 | 91.21 | 91.37 | 91.37 | 91.37 | 91.53 | 91.53 | 84.67 | 84.77 | 88.02 | 88.02 | 88.18 | 88.18 | 88.02 | 88.02 | 88.02 | 88.02 | 88.02 | 88.31 | 88.02 | 88.02 | 87.78 | 87.85 | 87.7 | 87.86 | 88.15 | 87.54 | 88.02 | 88.02 | 88.02 | 87.86 | 88.98 | 88.18 | 88.5 | 88.02 | 88.02 | 88.66 | 87.04 | 87.04 | 87.04 | 87.04 |
| **2** | CdF1 | 91.21 | 100 | 99.52 | 99.68 | 99.68 | 99.68 | 99.52 | 99.52 | 86.06 | 85.84 | 88.18 | 88.34 | 88.34 | 88.18 | 88.18 | 88.18 | 88.18 | 88.18 | 88.18 | 88.15 | 88.18 | 88.18 | 87.94 | 87.33 | 87.86 | 88.02 | 87.98 | 87.38 | 87.54 | 87.54 | 87.54 | 87.38 | 88.5 | 87.54 | 88.18 | 88.82 | 88.82 | 88.98 | 87.53 | 87.53 | 87.53 | 87.53 |
| **3** | MK648413 | 91.21 | 99.52 | 100 | 99.84 | 99.84 | 99.84 | 99.68 | 99.68 | 86.06 | 85.84 | 88.18 | 88.18 | 88.34 | 88.18 | 88.18 | 88.18 | 88.18 | 88.18 | 88.18 | 88.15 | 88.18 | 88.18 | 88.26 | 87.5 | 88.18 | 88.02 | 87.98 | 87.54 | 87.7 | 87.7 | 87.7 | 87.54 | 88.34 | 87.7 | 88.02 | 88.98 | 88.98 | 88.98 | 87.78 | 87.53 | 87.53 | 87.53 |
| **4** | KBF7 | 91.37 | 99.68 | 99.84 | 100 | 100 | 100 | 99.84 | 99.84 | 86.06 | 85.84 | 88.34 | 88.34 | 88.5 | 88.34 | 88.34 | 88.34 | 88.34 | 88.34 | 88.34 | 88.31 | 88.34 | 88.34 | 88.1 | 87.5 | 88.02 | 88.18 | 88.15 | 87.7 | 87.86 | 87.86 | 87.86 | 87.7 | 88.5 | 87.54 | 88.18 | 89.14 | 89.14 | 89.14 | 87.78 | 87.53 | 87.53 | 87.53 |
| **5** | KBF6 | 91.37 | 99.68 | 99.84 | 100 | 100 | 100 | 99.84 | 99.84 | 86.06 | 85.84 | 88.34 | 88.34 | 88.5 | 88.34 | 88.34 | 88.34 | 88.34 | 88.34 | 88.34 | 88.31 | 88.34 | 88.34 | 88.1 | 87.5 | 88.02 | 88.18 | 88.15 | 87.7 | 87.86 | 87.86 | 87.86 | 87.7 | 88.5 | 87.54 | 88.18 | 89.14 | 89.14 | 89.14 | 87.78 | 87.53 | 87.53 | 87.53 |
| **6** | AF132826 | 91.37 | 99.68 | 99.84 | 100 | 100 | 100 | 99.84 | 99.84 | 86.06 | 85.84 | 88.34 | 88.34 | 88.5 | 88.34 | 88.34 | 88.34 | 88.34 | 88.34 | 88.34 | 88.31 | 88.34 | 88.34 | 88.1 | 87.5 | 88.02 | 88.18 | 88.15 | 87.7 | 87.86 | 87.86 | 87.86 | 87.7 | 88.5 | 87.54 | 88.18 | 89.14 | 89.14 | 89.14 | 87.78 | 87.53 | 87.53 | 87.53 |
| **7** | CdF6 | 91.53 | 99.52 | 99.68 | 99.84 | 99.84 | 99.84 | 100 | 100 | 86.24 | 86.02 | 88.18 | 88.18 | 88.34 | 88.18 | 88.18 | 88.18 | 88.18 | 88.18 | 88.18 | 88.15 | 88.18 | 88.18 | 87.94 | 87.33 | 87.86 | 88.02 | 87.98 | 87.86 | 88.02 | 88.02 | 88.02 | 87.86 | 88.66 | 87.7 | 88.34 | 89.3 | 89.3 | 89.3 | 88.02 | 87.78 | 87.78 | 87.78 |
| **8** | KY678127 | 91.53 | 99.52 | 99.68 | 99.84 | 99.84 | 99.84 | 100 | 100 | 86.24 | 86.02 | 88.18 | 88.18 | 88.34 | 88.18 | 88.18 | 88.18 | 88.18 | 88.18 | 88.18 | 88.15 | 88.18 | 88.18 | 87.94 | 87.33 | 87.86 | 88.02 | 87.98 | 87.86 | 88.02 | 88.02 | 88.02 | 87.86 | 88.66 | 87.7 | 88.34 | 89.3 | 89.3 | 89.3 | 88.02 | 87.78 | 87.78 | 87.78 |
| **9** | KF18 | 84.67 | 86.06 | 86.06 | 86.06 | 86.06 | 86.06 | 86.24 | 86.24 | 100 | 99.82 | 85.89 | 85.37 | 85.71 | 85.54 | 85.54 | 85.54 | 85.54 | 85.54 | 85.54 | 85.37 | 85.54 | 85.54 | 85.54 | 85.54 | 85.37 | 85.37 | 85.19 | 85.02 | 84.67 | 85.02 | 85.02 | 85.02 | 86.59 | 86.24 | 86.24 | 86.93 | 86.93 | 85.89 | 83.62 | 83.37 | 83.37 | 83.37 |
| **10** | AF132833 | 84.77 | 85.84 | 85.84 | 85.84 | 85.84 | 85.84 | 86.02 | 86.02 | 99.82 | 100 | 86.02 | 85.48 | 85.84 | 85.66 | 85.66 | 85.66 | 85.66 | 85.66 | 85.66 | 85.66 | 85.66 | 85.66 | 85.66 | 85.66 | 85.48 | 85.48 | 85.47 | 85.13 | 84.77 | 85.13 | 85.13 | 85.13 | 86.74 | 86.38 | 86.38 | 87.1 | 87.1 | 86.02 | 83.62 | 83.37 | 83.37 | 83.37 |
| **11** | KX228549 | 88.02 | 88.18 | 88.18 | 88.34 | 88.34 | 88.34 | 88.18 | 88.18 | 85.89 | 86.02 | 100 | 99.36 | 99.52 | 99.52 | 99.68 | 99.68 | 99.68 | 99.68 | 99.68 | 99.67 | 99.68 | 99.68 | 99.52 | 99.65 | 99.36 | 99.52 | 99.5 | 94.57 | 94.57 | 94.89 | 94.89 | 94.73 | 94.41 | 92.81 | 93.13 | 92.17 | 92.17 | 92.49 | 91.44 | 91.69 | 91.69 | 91.44 |
| **12** | KX228548 | 88.02 | 88.34 | 88.18 | 88.34 | 88.34 | 88.34 | 88.18 | 88.18 | 85.37 | 85.48 | 99.36 | 100 | 99.52 | 99.68 | 99.68 | 99.68 | 99.68 | 99.68 | 99.68 | 99.67 | 99.68 | 99.68 | 99.52 | 99.65 | 99.36 | 99.52 | 99.5 | 94.41 | 94.41 | 94.73 | 94.73 | 94.57 | 94.25 | 92.65 | 92.97 | 92.33 | 92.33 | 92.81 | 91.93 | 91.93 | 91.93 | 91.69 |
| **13** | H3 | 88.18 | 88.34 | 88.34 | 88.5 | 88.5 | 88.5 | 88.34 | 88.34 | 85.71 | 85.84 | 99.52 | 99.52 | 100 | 99.68 | 99.84 | 99.84 | 99.84 | 99.84 | 99.84 | 99.83 | 99.84 | 99.84 | 99.68 | 99.83 | 99.52 | 99.68 | 99.67 | 94.41 | 94.41 | 94.73 | 94.73 | 94.57 | 94.25 | 92.65 | 92.97 | 92.01 | 92.01 | 92.65 | 91.93 | 92.18 | 92.18 | 91.93 |
| **14** | H1 | 88.18 | 88.18 | 88.18 | 88.34 | 88.34 | 88.34 | 88.18 | 88.18 | 85.54 | 85.66 | 99.52 | 99.68 | 99.68 | 100 | 99.84 | 99.84 | 99.84 | 99.84 | 99.84 | 99.83 | 99.84 | 99.84 | 99.68 | 99.83 | 99.52 | 99.68 | 99.67 | 94.57 | 94.57 | 94.89 | 94.89 | 94.73 | 94.41 | 92.81 | 93.13 | 92.17 | 92.17 | 92.65 | 91.93 | 91.93 | 91.93 | 91.69 |
| **15** | KC503261 | 88.02 | 88.18 | 88.18 | 88.34 | 88.34 | 88.34 | 88.18 | 88.18 | 85.54 | 85.66 | 99.68 | 99.68 | 99.84 | 99.84 | 100 | 100 | 100 | 100 | 100 | 100 | 100 | 100 | 99.84 | 100 | 99.68 | 99.84 | 99.83 | 94.57 | 94.57 | 94.89 | 94.89 | 94.73 | 94.41 | 92.81 | 93.13 | 92.17 | 92.17 | 92.81 | 91.93 | 92.18 | 92.18 | 91.93 |
| **16** | KY678117 | 88.02 | 88.18 | 88.18 | 88.34 | 88.34 | 88.34 | 88.18 | 88.18 | 85.54 | 85.66 | 99.68 | 99.68 | 99.84 | 99.84 | 100 | 100 | 100 | 100 | 100 | 100 | 100 | 100 | 99.84 | 100 | 99.68 | 99.84 | 99.83 | 94.57 | 94.57 | 94.89 | 94.89 | 94.73 | 94.41 | 92.81 | 93.13 | 92.17 | 92.17 | 92.81 | 91.93 | 92.18 | 92.18 | 91.93 |
| **17** | H2 | 88.02 | 88.18 | 88.18 | 88.34 | 88.34 | 88.34 | 88.18 | 88.18 | 85.54 | 85.66 | 99.68 | 99.68 | 99.84 | 99.84 | 100 | 100 | 100 | 100 | 100 | 100 | 100 | 100 | 99.84 | 100 | 99.68 | 99.84 | 99.83 | 94.57 | 94.57 | 94.89 | 94.89 | 94.73 | 94.41 | 92.81 | 93.13 | 92.17 | 92.17 | 92.81 | 91.93 | 92.18 | 92.18 | 91.93 |
| **18** | Rm_CF4 | 88.02 | 88.18 | 88.18 | 88.34 | 88.34 | 88.34 | 88.18 | 88.18 | 85.54 | 85.66 | 99.68 | 99.68 | 99.84 | 99.84 | 100 | 100 | 100 | 100 | 100 | 100 | 100 | 100 | 99.84 | 100 | 99.68 | 99.84 | 99.83 | 94.57 | 94.57 | 94.89 | 94.89 | 94.73 | 94.41 | 92.81 | 93.13 | 92.17 | 92.17 | 92.81 | 91.93 | 92.18 | 92.18 | 91.93 |
| **19** | Rm_CF5 | 88.02 | 88.18 | 88.18 | 88.34 | 88.34 | 88.34 | 88.18 | 88.18 | 85.54 | 85.66 | 99.68 | 99.68 | 99.84 | 99.84 | 100 | 100 | 100 | 100 | 100 | 100 | 100 | 100 | 99.84 | 100 | 99.68 | 99.84 | 99.83 | 94.57 | 94.57 | 94.89 | 94.89 | 94.73 | 94.41 | 92.81 | 93.13 | 92.17 | 92.17 | 92.81 | 91.93 | 92.18 | 92.18 | 91.93 |
| **20** | MG983831 | 88.31 | 88.15 | 88.15 | 88.31 | 88.31 | 88.31 | 88.15 | 88.15 | 85.37 | 85.66 | 99.67 | 99.67 | 99.83 | 99.83 | 100 | 100 | 100 | 100 | 100 | 100 | 100 | 100 | 99.83 | 100 | 99.67 | 99.83 | 99.83 | 94.49 | 94.49 | 94.82 | 94.82 | 94.66 | 94.16 | 92.49 | 92.82 | 92.15 | 92.15 | 92.65 | 91.62 | 91.88 | 91.88 | 91.62 |
| **21** | KY678118 | 88.02 | 88.18 | 88.18 | 88.34 | 88.34 | 88.34 | 88.18 | 88.18 | 85.54 | 85.66 | 99.68 | 99.68 | 99.84 | 99.84 | 100 | 100 | 100 | 100 | 100 | 100 | 100 | 100 | 99.84 | 100 | 99.68 | 99.84 | 99.83 | 94.57 | 94.57 | 94.89 | 94.89 | 94.73 | 94.41 | 92.81 | 93.13 | 92.17 | 92.17 | 93.16 | 92.59 | 92.77 | 92.77 | 92.59 |
| **22** | KY678120 | 88.02 | 88.18 | 88.18 | 88.34 | 88.34 | 88.34 | 88.18 | 88.18 | 85.54 | 85.66 | 99.68 | 99.68 | 99.84 | 99.84 | 100 | 100 | 100 | 100 | 100 | 100 | 100 | 100 | 99.84 | 100 | 99.68 | 99.84 | 99.83 | 94.57 | 94.57 | 94.89 | 94.89 | 94.73 | 94.41 | 92.81 | 93.13 | 92.17 | 92.17 | 93.16 | 92.59 | 92.77 | 92.77 | 92.59 |
| **23** | MK648412 | 87.78 | 87.94 | 88.26 | 88.1 | 88.1 | 88.1 | 87.94 | 87.94 | 85.54 | 85.66 | 99.52 | 99.52 | 99.68 | 99.68 | 99.84 | 99.84 | 99.84 | 99.84 | 99.84 | 99.83 | 99.84 | 99.84 | 100 | 100 | 99.84 | 99.68 | 99.66 | 94.53 | 94.37 | 94.69 | 94.69 | 94.69 | 94.21 | 92.93 | 92.93 | 91.96 | 91.96 | 92.6 | 91.93 | 92.18 | 92.18 | 91.93 |
| **24** | MF458973 | 87.85 | 87.33 | 87.5 | 87.5 | 87.5 | 87.5 | 87.33 | 87.33 | 85.54 | 85.66 | 99.65 | 99.65 | 99.83 | 99.83 | 100 | 100 | 100 | 100 | 100 | 100 | 100 | 100 | 100 | 100 | 99.83 | 99.83 | 99.82 | 94.62 | 94.44 | 94.79 | 94.79 | 94.79 | 94.27 | 93.06 | 92.88 | 92.01 | 92.01 | 92.88 | 91.93 | 92.18 | 92.18 | 91.93 |
| **25** | KT906181 | 87.7 | 87.86 | 88.18 | 88.02 | 88.02 | 88.02 | 87.86 | 87.86 | 85.37 | 85.48 | 99.36 | 99.36 | 99.52 | 99.52 | 99.68 | 99.68 | 99.68 | 99.68 | 99.68 | 99.67 | 99.68 | 99.68 | 99.84 | 99.83 | 100 | 99.84 | 99.83 | 94.25 | 94.25 | 94.57 | 94.57 | 94.41 | 94.09 | 92.81 | 92.81 | 91.85 | 91.85 | 92.49 | 91.69 | 91.93 | 91.93 | 91.69 |
| **26** | KP143546 | 87.86 | 88.02 | 88.02 | 88.18 | 88.18 | 88.18 | 88.02 | 88.02 | 85.37 | 85.48 | 99.52 | 99.52 | 99.68 | 99.68 | 99.84 | 99.84 | 99.84 | 99.84 | 99.84 | 99.83 | 99.84 | 99.84 | 99.68 | 99.83 | 99.84 | 100 | 100 | 94.41 | 94.41 | 94.73 | 94.73 | 94.57 | 94.25 | 92.65 | 92.97 | 92.01 | 92.01 | 92.65 | 91.69 | 91.93 | 91.93 | 91.69 |
| **27** | MG983832 | 88.15 | 87.98 | 87.98 | 88.15 | 88.15 | 88.15 | 87.98 | 87.98 | 85.19 | 85.47 | 99.5 | 99.5 | 99.67 | 99.67 | 99.83 | 99.83 | 99.83 | 99.83 | 99.83 | 99.83 | 99.83 | 99.83 | 99.66 | 99.82 | 99.83 | 100 | 100 | 94.32 | 94.32 | 94.66 | 94.66 | 94.49 | 93.99 | 92.32 | 92.65 | 91.99 | 91.99 | 92.49 | 91.36 | 91.62 | 91.62 | 91.36 |
| **28** | KC503255 | 87.54 | 87.38 | 87.54 | 87.7 | 87.7 | 87.7 | 87.86 | 87.86 | 85.02 | 85.13 | 94.57 | 94.41 | 94.41 | 94.57 | 94.57 | 94.57 | 94.57 | 94.57 | 94.57 | 94.49 | 94.57 | 94.57 | 94.53 | 94.62 | 94.25 | 94.41 | 94.32 | 100 | 99.04 | 99.36 | 99.36 | 99.52 | 92.97 | 91.21 | 91.53 | 91.37 | 91.37 | 91.21 | 90.95 | 90.71 | 90.71 | 90.46 |
| **29** | QLF6 | 88.02 | 87.54 | 87.7 | 87.86 | 87.86 | 87.86 | 88.02 | 88.02 | 84.67 | 84.77 | 94.57 | 94.41 | 94.41 | 94.57 | 94.57 | 94.57 | 94.57 | 94.57 | 94.57 | 94.49 | 94.57 | 94.57 | 94.37 | 94.44 | 94.25 | 94.41 | 94.32 | 99.04 | 100 | 99.68 | 99.68 | 99.52 | 92.65 | 91.21 | 91.53 | 91.05 | 91.05 | 91.53 | 91.2 | 90.95 | 90.95 | 90.71 |
| **30** | QLF1 | 88.02 | 87.54 | 87.7 | 87.86 | 87.86 | 87.86 | 88.02 | 88.02 | 85.02 | 85.13 | 94.89 | 94.73 | 94.73 | 94.89 | 94.89 | 94.89 | 94.89 | 94.89 | 94.89 | 94.82 | 94.89 | 94.89 | 94.69 | 94.79 | 94.57 | 94.73 | 94.66 | 99.36 | 99.68 | 100 | 100 | 99.84 | 92.97 | 91.21 | 91.53 | 91.05 | 91.05 | 91.53 | 91.2 | 90.95 | 90.95 | 90.71 |
| **31** | QLF4 | 88.02 | 87.54 | 87.7 | 87.86 | 87.86 | 87.86 | 88.02 | 88.02 | 85.02 | 85.13 | 94.89 | 94.73 | 94.73 | 94.89 | 94.89 | 94.89 | 94.89 | 94.89 | 94.89 | 94.82 | 94.89 | 94.89 | 94.69 | 94.79 | 94.57 | 94.73 | 94.66 | 99.36 | 99.68 | 100 | 100 | 99.84 | 92.97 | 91.21 | 91.53 | 91.05 | 91.05 | 91.53 | 91.2 | 90.95 | 90.95 | 90.71 |
| **32** | AF132827 | 87.86 | 87.38 | 87.54 | 87.7 | 87.7 | 87.7 | 87.86 | 87.86 | 85.02 | 85.13 | 94.73 | 94.57 | 94.57 | 94.73 | 94.73 | 94.73 | 94.73 | 94.73 | 94.73 | 94.66 | 94.73 | 94.73 | 94.69 | 94.79 | 94.41 | 94.57 | 94.49 | 99.52 | 99.52 | 99.84 | 99.84 | 100 | 92.81 | 91.05 | 91.37 | 90.89 | 90.89 | 91.37 | 91.2 | 90.95 | 90.95 | 90.71 |
| **33** | KX228542 | 88.98 | 88.5 | 88.34 | 88.5 | 88.5 | 88.5 | 88.66 | 88.66 | 86.59 | 86.74 | 94.41 | 94.25 | 94.25 | 94.41 | 94.41 | 94.41 | 94.41 | 94.41 | 94.41 | 94.16 | 94.41 | 94.41 | 94.21 | 94.27 | 94.09 | 94.25 | 93.99 | 92.97 | 92.65 | 92.97 | 92.97 | 92.81 | 100 | 98.24 | 98.4 | 94.25 | 94.25 | 94.57 | 93.15 | 92.91 | 92.91 | 92.67 |
| **34** | MK648411 | 88.18 | 87.54 | 87.7 | 87.54 | 87.54 | 87.54 | 87.7 | 87.7 | 86.24 | 86.38 | 92.81 | 92.65 | 92.65 | 92.81 | 92.81 | 92.81 | 92.81 | 92.81 | 92.81 | 92.49 | 92.81 | 92.81 | 92.93 | 93.06 | 92.81 | 92.65 | 92.32 | 91.21 | 91.21 | 91.21 | 91.21 | 91.05 | 98.24 | 100 | 99.2 | 93.61 | 93.61 | 94.09 | 93.4 | 93.15 | 93.15 | 92.91 |
| **35** | KY678123 | 88.5 | 88.18 | 88.02 | 88.18 | 88.18 | 88.18 | 88.34 | 88.34 | 86.24 | 86.38 | 93.13 | 92.97 | 92.97 | 93.13 | 93.13 | 93.13 | 93.13 | 93.13 | 93.13 | 92.82 | 93.13 | 93.13 | 92.93 | 92.88 | 92.81 | 92.97 | 92.65 | 91.53 | 91.53 | 91.53 | 91.53 | 91.37 | 98.4 | 99.2 | 100 | 94.09 | 94.09 | 94.89 | 93.64 | 93.4 | 93.4 | 93.15 |
| **36** | HF7 | 88.02 | 88.82 | 88.98 | 89.14 | 89.14 | 89.14 | 89.3 | 89.3 | 86.93 | 87.1 | 92.17 | 92.33 | 92.01 | 92.17 | 92.17 | 92.17 | 92.17 | 92.17 | 92.17 | 92.15 | 92.17 | 92.17 | 91.96 | 92.01 | 91.85 | 92.01 | 91.99 | 91.37 | 91.05 | 91.05 | 91.05 | 90.89 | 94.25 | 93.61 | 94.09 | 100 | 100 | 95.37 | 93.89 | 93.64 | 93.64 | 93.4 |
| **37** | HF11 | 88.02 | 88.82 | 88.98 | 89.14 | 89.14 | 89.14 | 89.3 | 89.3 | 86.93 | 87.1 | 92.17 | 92.33 | 92.01 | 92.17 | 92.17 | 92.17 | 92.17 | 92.17 | 92.17 | 92.15 | 92.17 | 92.17 | 91.96 | 92.01 | 91.85 | 92.01 | 91.99 | 91.37 | 91.05 | 91.05 | 91.05 | 90.89 | 94.25 | 93.61 | 94.09 | 100 | 100 | 95.37 | 93.89 | 93.64 | 93.64 | 93.4 |
| **c** | KC503259 | 88.66 | 88.98 | 88.98 | 89.14 | 89.14 | 89.14 | 89.3 | 89.3 | 85.89 | 86.02 | 92.49 | 92.81 | 92.65 | 92.65 | 92.81 | 92.81 | 92.81 | 92.81 | 92.81 | 92.65 | 93.16 | 93.16 | 92.6 | 92.88 | 92.49 | 92.65 | 92.49 | 91.21 | 91.53 | 91.53 | 91.53 | 91.37 | 94.57 | 94.09 | 94.89 | 95.37 | 95.37 | 100 | 99.51 | 99.67 | 99.67 | 99.51 |
| **39** | JQ625684 | 87.04 | 87.53 | 87.78 | 87.78 | 87.78 | 87.78 | 88.02 | 88.02 | 83.62 | 83.62 | 91.44 | 91.93 | 91.93 | 91.93 | 91.93 | 91.93 | 91.93 | 91.93 | 91.93 | 91.62 | 92.59 | 92.59 | 91.93 | 91.93 | 91.69 | 91.69 | 91.36 | 90.95 | 91.2 | 91.2 | 91.2 | 91.2 | 93.15 | 93.4 | 93.64 | 93.89 | 93.89 | 99.51 | 100 | 99.84 | 99.84 | 99.67 |
| **40** | JQ625683 | 87.04 | 87.53 | 87.53 | 87.53 | 87.53 | 87.53 | 87.78 | 87.78 | 83.37 | 83.37 | 91.69 | 91.93 | 92.18 | 91.93 | 92.18 | 92.18 | 92.18 | 92.18 | 92.18 | 91.88 | 92.77 | 92.77 | 92.18 | 92.18 | 91.93 | 91.93 | 91.62 | 90.71 | 90.95 | 90.95 | 90.95 | 90.95 | 92.91 | 93.15 | 93.4 | 93.64 | 93.64 | 99.67 | 99.84 | 100 | 100 | 99.84 |
| **41** | JF758630 | 87.04 | 87.53 | 87.53 | 87.53 | 87.53 | 87.53 | 87.78 | 87.78 | 83.37 | 83.37 | 91.69 | 91.93 | 92.18 | 91.93 | 92.18 | 92.18 | 92.18 | 92.18 | 92.18 | 91.88 | 92.77 | 92.77 | 92.18 | 92.18 | 91.93 | 91.93 | 91.62 | 90.71 | 90.95 | 90.95 | 90.95 | 90.95 | 92.91 | 93.15 | 93.4 | 93.64 | 93.64 | 99.67 | 99.84 | 100 | 100 | 99.84 |
| **42** | JF758636 | 87.04 | 87.53 | 87.53 | 87.53 | 87.53 | 87.53 | 87.78 | 87.78 | 83.37 | 83.37 | 91.44 | 91.69 | 91.93 | 91.69 | 91.93 | 91.93 | 91.93 | 91.93 | 91.93 | 91.62 | 92.59 | 92.59 | 91.93 | 91.93 | 91.69 | 91.69 | 91.36 | 90.46 | 90.71 | 90.71 | 90.71 | 90.71 | 92.67 | 92.91 | 93.15 | 93.4 | 93.4 | 99.51 | 99.67 | 99.84 | 99.84 | 100 |

Percent identity analyses was performed using Clustal Omega multiple sequence analyses tool [30]; <https://www.ebi.ac.uk/Tools/msa/clustalo/>). The values represent % identities between two pairs of sequences with corresponding numbers. The analysis involved 42 *cox*1 sequences. These included the three Kenyan *R. microplus* haplotype sequences (H1-H3), *cox*1 sequences of *R. microplus* reference ticks from Cameroon (CF4 and CF5), from Laos (HF7 and H11), *R. australis* (QLDF1, QLDF4, QLDF6), *R. decoloratus* from Kenya (KBF6 and KBF7) and Cameroon (CdF1 and CdF6), all analysed in this study. Their similarities were compared to three GenBank *R. microplus* reference sequences from Cameroon (MK648412, MG983832, MG983831), one from DR Congo (MF45873), South Africa (KY678117), Benin (KY678120) and Madagascar (KY678118), USA (KP143546), Colombia (KT906181) and Philippines (KX228548). A *R. microplus* sequence from Kenya (KX228549), two of *R. australis* (KC503255 and AF132827), five *R. microplus* sequences from China (KC503259, JQ625684, JQ625683, JF758630, JF758636), three *R. annulatus* sequences from India (KX228542), Cameroon (MK648411) and Burkina Faso (KY678123), four *R. decoloratus* sequences from South Africa (KY678130, AF132826), Cameroon (MK648413) and Burkina Faso (KY678127) and one *R. appendiculatus* sequence (AF132833) were also compared.
