# Supplementary material for: Molecular evidence confirms occurrence of Rhipicephalus microplus Clade A in Kenya and sub-Saharan Africa
Source: Parasit Vectors. 2020 Aug 27;13:432. doi: 10.1186/s13071-020-04266-0 (PMC7453536; doi:10.1186/s13071-020-04266-0)
Supplement: Supplementary file 4 — Additional file 4: Table S4. Mitochondrial DNA (mtDNA) sequences percent (%) identity matrix. [file 13071_2020_4266_MOESM4_ESM.docx]

**Additional file 4: Table S4. Mitochondrial DNA (mtDNA) sequences percent (%) identity matrix**

| **No** | **Tick species** | **Sequence** | **1** | **2** | **3** | **4** | **5** | **6** | **7** | **8** | **9** | **10** | **11** | **12** | **13** | **14** | **15** | **16** | **17** |
| --- | --- | --- | --- | --- | --- | --- | --- | --- | --- | --- | --- | --- | --- | --- | --- | --- | --- | --- | --- |
| **1** | *H. longicornis* | MK450606 | 100 | 72.93 | 73 | 73.99 | 75.34 | 73.71 | 73.35 | 73.87 | 75.12 | 74.05 | 74.04 | 73.05 | 73.45 | 73.31 | 73.42 | 73.4 | 73.39 |
| **2** | *R. sanguineus* | AF081829 | 72.93 | 100 | 89.2 | 83.36 | 83.28 | 82.59 | 82.25 | 82.63 | 82.29 | 82.16 | 82.16 | 81.41 | 81.91 | 81.79 | 81.89 | 81.88 | 81.91 |
| **3** | *R. turanicus* | KY996841 | 73 | 89.2 | 100 | 82.99 | 82.86 | 82.37 | 81.72 | 82.04 | 82.19 | 81.79 | 81.77 | 81.13 | 81.57 | 81.57 | 81.55 | 81.49 | 81.5 |
| **4** | *R. appendiculatus* (Kenya) | KF10 | 73.99 | 83.36 | 82.99 | 100 | 99.18 | 83.49 | 83.33 | 82.81 | 83.69 | 82.59 | 82.57 | 82.79 | 82.87 | 82.85 | 82.89 | 82.84 | 82.83 |
| **5** | *R. appendiculatus* (Zimbabwe) | KC503257 | 75.34 | 83.28 | 82.86 | 99.18 | 100 | 83.22 | 82.9 | 82.89 | 82.89 | 83.68 | 83.68 | 82.51 | 82.39 | 82.82 | 82.79 | 82.76 | 82.69 |
| **6** | *R. geigyi* (Burkina Faso) | KC503263 | 73.71 | 82.59 | 82.37 | 83.49 | 83.22 | 100 | 90.71 | 90.54 | 87.85 | 87.32 | 87.32 | 87.13 | 87.21 | 87.41 | 87.36 | 87.32 | 87.26 |
| **7** | *R. decoloratus* (S. Africa) | KY457525 | 73.35 | 82.25 | 81.72 | 83.33 | 82.9 | 90.71 | 100 | 99.13 | 87.52 | 87.35 | 87.32 | 87.08 | 87.06 | 87.16 | 87.19 | 87.23 | 87.2 |
| **8** | *R. decoloratus* (Kenya) | KBF6 | 73.87 | 82.63 | 82.04 | 82.81 | 82.89 | 90.54 | 99.13 | 100 | 87.39 | 87.18 | 87.15 | 87.24 | 87.17 | 86.73 | 86.76 | 86.81 | 86.22 |
| **9** | *R. annulatus* (Romania) | KC503256 | 75.12 | 82.29 | 82.19 | 83.69 | 82.89 | 87.85 | 87.52 | 87.39 | 100 | 94.25 | 94.27 | 95.59 | 93.92 | 94.45 | 94.34 | 94.29 | 94.27 |
| **10** | *R. microplus* (Kenya) | KF13 | 74.05 | 82.16 | 81.79 | 82.59 | 83.68 | 87.32 | 87.35 | 87.18 | 94.25 | 100 | 99.97 | 94.34 | 96.07 | 98.97 | 99.58 | 99.79 | 99.83 |
| **11** | *R. microplus* (Kenya) | KSF2 | 74.04 | 82.16 | 81.77 | 82.57 | 83.68 | 87.32 | 87.32 | 87.15 | 94.27 | 99.97 | 100 | 94.34 | 96.06 | 98.97 | 99.59 | 99.79 | 99.84 |
| **12** | *R. microplus* (China) | KC503259 | 73.05 | 81.41 | 81.13 | 82.79 | 82.51 | 87.13 | 87.08 | 87.24 | 95.59 | 94.34 | 94.34 | 100 | 94.07 | 94.53 | 94.22 | 94.19 | 94.21 |
| **13** | *R. microplus* (Australia) | KC503255 | 73.45 | 81.91 | 81.57 | 82.87 | 82.39 | 87.21 | 87.06 | 87.17 | 93.92 | 96.07 | 96.06 | 94.07 | 100 | 95.87 | 96.07 | 96.01 | 96.03 |
| **14** | *R. microplus* (India) | MK234703 | 73.31 | 81.79 | 81.57 | 82.85 | 82.82 | 87.41 | 87.16 | 86.73 | 94.45 | 98.97 | 98.97 | 94.53 | 95.87 | 100 | 99.34 | 98.99 | 99.03 |
| **15** | *R. microplus* (Cambodia) | KC503260 | 73.42 | 81.89 | 81.55 | 82.89 | 82.79 | 87.36 | 87.19 | 86.76 | 94.34 | 99.58 | 99.59 | 94.22 | 96.07 | 99.34 | 100 | 99.56 | 99.6 |
| **16** | *R. microplus* (Brazil) | KC503261 | 73.4 | 81.88 | 81.49 | 82.84 | 82.76 | 87.32 | 87.23 | 86.81 | 94.29 | 99.79 | 99.79 | 94.19 | 96.01 | 98.99 | 99.56 | 100 | 99.87 |
| **17** | R. microplus (USA) | KP143546 | 73.39 | 81.91 | 81.5 | 82.83 | 82.69 | 87.26 | 87.2 | 86.22 | 94.27 | 99.83 | 99.84 | 94.21 | 96.03 | 99.03 | 99.6 | 99.87 | 100 |

Percent identity analyses of 17 mtDNA sequences was performed using Clustal Omega multiple sequence analyses tool [30]; <https://www.ebi.ac.uk/Tools/msa/clustalo/>). Values represent percent identities between a pair of sequences matching to corresponding numbers. Four mtDNA genomes analysed in this study are highlighted (*R. microplus* KF13 and KSF2, *R. appendiculatus* KF10 and *R. decoloratus* KBF6). Six *R. microplus* genomes and one genome each for *R. annulatus, R. geigyi, R. sanguineus*, *R. turanicus* and *H. longicornis* from GenBank were included. A partial mtDNA genome of *R. appendiculatus* (KC503257) from Zimbabwe and an unverified *R. decoloratus* genome (KY457525) from South Africa available in GenBank were used to compare the genome sequences of *R. appendiculatus* and *R. decoloratus*.
